# Decoding pancreatic endocrine cell differentiation and β cell regeneration in zebrafish

**DOI:** 10.1126/sciadv.adf5142

**Published:** 2023-08-18

**Authors:** Jiarui Mi, Ka-Cheuk Liu, Olov Andersson

**Affiliations:** Department of Cell and Molecular Biology, Karolinska Institutet, 17177 Stockholm, Sweden.

## Abstract

In contrast to mice, zebrafish have an exceptional yet elusive ability to replenish lost β cells in adulthood. Understanding this framework would provide mechanistic insights for β cell regeneration, which may be extrapolated to humans. Here, we characterize a *krt4*-expressing ductal cell type, which is distinct from the putative Notch-responsive cells, showing neogenic competence and giving rise to the majority of endocrine cells during postembryonic development. Furthermore, we demonstrate a marked ductal remodeling process featuring a Notch-responsive to *krt4*^+^ luminal duct transformation during late development, indicating several origins of *krt4*^+^ ductal cells displaying similar transcriptional patterns. Single-cell transcriptomics upon a series of time points during β cell regeneration unveil a previously unrecognized *dlb*^+^ transitional endocrine precursor cell, distinct regulons, and a differentiation trajectory involving cellular shuffling through differentiation and dedifferentiation dynamics. These results establish a model of zebrafish pancreatic endocrinogenesis and highlight key values of zebrafish for translational studies of β cell regeneration.

## Teaser

*krt4^+^* ductal progenitors and *dlb^+^* endocrine precursors serve as a direct origin for β cells in the zebrafish pancreas.

## INTRODUCTION

During murine and human embryonic development of the pancreas, the ductal cells reside in the trunk of the branched epithelium and can up-regulate the transcription factors *Neurog3* and *Fev* to induce early and late endocrine progenitor cells, respectively (*1*–*5*). The late progenitor cells are dedicated to specific committed endocrine cell types based on a sophisticated balance among a wide range of lineage-specific transcription factors for final cell fate acquisition (*3*, *6*–*8*). This differentiation process is sustained until the neonatal stage and markedly declines soon after birth. In mouse, upon β cell injury, which can be induced by numerous genetic and pharmacological strategies, the proliferation of remnant β cells serves as the primary source of regenerative β cells, indicated by pulse-chase experiment and lineage fate mapping studies (*9*, *10*). Notably, the induction of massive β cell loss, overexpression of a key transcription factor cocktail using genetic approaches, or inhibition of cell differentiation blockades can stimulate a variety of cell types to up-regulate insulin expression, highlighting the astonishing cell plasticity in a broad range of cells for reprogramming toward pancreatic β cell fate (*11*–*19*). Some studies suggest that the pancreatic duct might harbor a latent progenitor population to give rise to insulin-producing cells under diseased conditions in adults (*20*–*24*). However, this is still under debate due to a lack of reliable ductal cell–specific lineage tracing tools in mouse models and speculated endocrine cell contaminations in human samples (*20*, *25*, *26*).

Zebrafish, a widely used model organism for regeneration studies, can fully recover from extreme β cell loss in both larval and adult stages within 1 month (*27*, *28*). Lineage tracing experiments with temporal labeling of β cells indicated that although proliferation of preexisting β cells can be accelerated by drug treatment, there are other sources contributing to the restoration of the majority of insulin-producing cells upon β cell ablation (*29*, *30*). The main sources of developing β cells and regenerative insulin–producing cells have yet to be determined by genetic fate mapping. However, prevailing evidence from lineage tracing or similar studies using transgenic zebrafish strains indicates that a small fraction of endocrine cells in the secondary islets can be formed from Notch-responsive cells (also referred to as intrapancreatic ductal cells or centroacinar cells) (*31*–*33*); however, they do not explain the total contribution for the endocrine pancreas. Thus, broader or different ductal/progenitor cell–specific Cre lines are needed to expand the genetic fate mapping to include the majority of endocrine cell formation (*30*, *34*–*38*). Moreover, recent studies suggested that *sst1.1^+^* endocrine cells might convert to insulin-producing cells upon β cell ablation (*37*, *39*). However, evidence from lineage tracing is still lacking.

During zebrafish development, the early pancreatic endocrine cells arise from the dorsal pancreatic bud and culminate at around 36 hours after fertilization (*40*). The ventral pancreatic bud subsequently engulfs the dorsal bud and gives rise to exocrine tissues and additional endocrine cells that coalesce with the principal islet in the head of the pancreas (*40*). At around 5 days postfertilization (dpf), in parallel with the start of food intake, secondary islets in the pancreatic tail region occasionally appear, and more β cells will progressively form in both the principal and secondary islets through adulthood as a response to increased nutrient consumption (*41*). Using pharmacological and genetic approaches, researchers have shown that the downregulation of Notch signaling can lead to a substantial increase in β cells in the secondary islets (*31*, *42*, *43*). Lineage fate mapping experiments using the *tp1* (a Notch-responsive viral element) promoter to drive CreERT2 commonly label around 75% of Notch-responsive cells but fail to label the majority of endocrine cells in the anterior region of the principal and the secondary islets, indicating that other surrogate cell sources might exist with a unique reprogramming mechanism underlying the differentiation process (*32*).

To resolve this apparent disparity, we combined a variety of state-of-the-art tools including single-cell RNA sequencing (RNA-seq), newly generated knock-in and transgenic lines for lineage fate mapping, and targeted cell ablation to characterize a previously undefined ductal cell type, i.e., *krt4^+^* ductal cells that function as the ductal progenitors of endocrine cells. This type of ductal cell has a transcriptional signature, morphological profile, and organization distinct from the putative Notch-responsive ductal cells. Briefly, these *krt4^+^* ductal cells display an alternative Notch-signaling state and assemble together to gradually form luminal structures throughout the pancreas. These ductal cells, coupled with a subset of *neurod1^+^* endocrine cells, can convert to bihormonal (also known as *ins^+^*&*sst1.1^+^* hybrid) cells after β cell ablation. Moreover, the *krt4*^*+*.^ luminal duct has several independent origins and eventually forms large connecting tubular structures, with a common transcriptional pattern in the process of ductal tree morphogenesis. Last, by sequencing the *krt4* lineage at the single-cell level in the basal state and after β cell ablation, we uncovered *dlb^+^* endocrine progenitor cells and several transitional cell states along the newly defined differentiation trajectory toward bihormonal and mature cells. By harnessing the up-regulated molecular events in the transitional cell clusters, e.g., by inhibiting the phosphatidylinositol 3-kinase (PI3K)–AKT–mammalian target of rapamycin (mTOR) pathway, we can induce *krt4^+^* duct–derived insulin-producing cells. In all, we generate a blueprint of zebrafish endocrinogenesis and put forward a redefined model of endocrine cellular origin that encompasses an emerging concept linking it with ductal remodeling. Our single-cell analyses show that endocrinogenesis is largely conserved when comparing adult zebrafish with embryonic mouse and human and uncover previously unknown key molecular events governing β cell formation and regeneration.

## RESULTS

### Single-cell transcriptomics identifies ductal heterogeneity and *krt4*^+^ duct in adult zebrafish

**Fig. 1.**
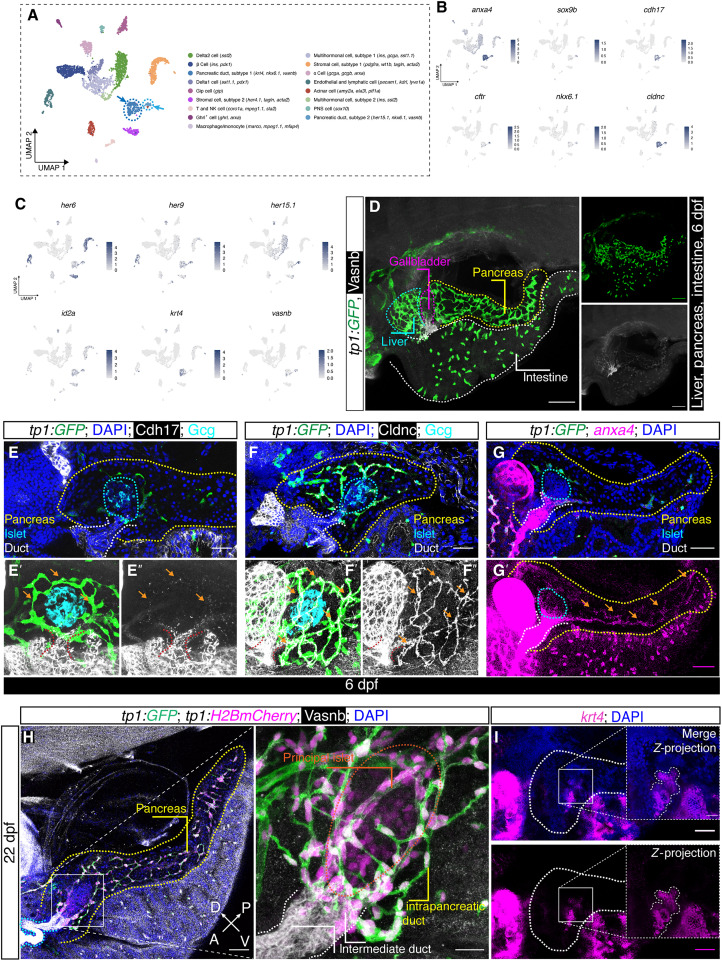
Ductal heterogeneity in the adult zebrafish pancreas. (**A**) Uniform Manifold Approximation and Projection (UMAP) plot showing the different cell clusters. The classifications were based on known marker genes highly expressed in each cell type in the zebrafish pancreas. The two clusters of pancreatic ductal cell types are denoted by dashed lines and arrows. NK, natural killer. (**B** and **C**) Feature plot shown in UMAP embedding of selected ductal markers (B), Notch and BMP downstream genes enriched in pancreatic ducts, and novel ductal markers (C). (**D**) Immunostaining using a Vasnb antibody in *Tg(tp1:EGFP)* transgenic larva showing the ducts in the hepatopancreatic biliary system. The dashed lines outline the organs in different colors, i.e., pancreas (yellow), gallbladder (magenta), liver (cyan), and intestine (white). Scale bars, 200 μm. (**E** to **G**) Representative confocal images of immunostaining of Cdh17 and Cldnc in *Tg(tp1:EGFP)* larvae (E) and (F) and in situ hybridization for *anxa4* in *Tg(tp1:EGFP)* larvae (G) with *Z*-projection (E′) and (E″), (F′) and (F″), and (G′). The pancreas (yellow), islet (cyan), and non–Notch responsive ducts (white) were outlined by dashed lines in different colors. The Notch-responsive intrapancreatic ducts are indicated by arrows (orange). Scale bars, 200 and 40 μm (magnification). (**H**) Double transgenic for *tp1:EGFP* and *tp1:H2BmCherry* with anti-Vasnb staining showing the labeling pattern in the duct and islets in the larvae pancreas. The pancreata, extrapancreatic duct, intermediate duct, and islet are highlighted with yellow, cyan, white, and orange dashed lines, respectively. Scale bars, 200 μm (left) and 40 μm (right). A, anterior; P, posterior; D, dorsal; V, ventral. (**I**) In situ hybridization for *krt4* showing the extrapancreatic-to-intermediate duct system with bifurcation in 6 dpf larva. The insets are magnified *Z*-projections with white dashed lines outlining the tree-like structure of the intermediate duct. Scale bars, 200 and 40 μm (insets).

For a preliminary view of the cellular composition of the islet and the surrounding niche, we first analyzed one public single-cell RNA-seq dataset of the adult zebrafish principal islet (*44*). After filtering and quality control, we identified 17 clusters, two of which showed expression of various well-known ductal markers including *sox9b*, *nkx6.1*, *cftr*, and *anxa4* (Fig. 1, A and B). We noted that these two clusters differ in terms of Notch-signaling responsive genes, with the larger cluster expressing *her6* and *her9* to a low extent, while the smaller cluster preferentially expressed *her15.1*. Both clusters are *id2a*^+^, indicating that they are both bone morphogenetic protein (BMP) responsive (Fig. 1C). Next, we demonstrated that *epcam*, *cldn7b*, *cd9b*, *cx30.3*, and *onecut1* were expressed in both types of ducts, while *clcn1b* and *igf2a* presented highest expression in *her15.1^+^* duct (fig. S1, A to H). We also identified *vasnb*, *cdh17*, and *cldnc*, which code for membrane-bound proteins, as previously undefined pan-ductal marker genes (Fig. 1, B and C). Immunostaining for Vasnb, Cdh17, and Cldnc, as well as hybridization chain reaction (HCR) 3.0 in situ immunofluorescence of *anxa4* in the *Tg(Tp1bglob:eGFP)^um14Tg^* [abbreviated as *Tg(tp1:EGFP)*] larvae at 6 dpf, displayed two types of morphologically distinct ductal cells with or without green fluorescent protein (GFP) labeling, consolidating the prior in silico analyses (Fig. 1, D to G) (*45*, *46*).

Immunostaining of *Tg(tp1:EGFP);Tg(tp1:H2BmCherry)* larvae at 22 dpf showed that the ducts in the anterior region of the pancreas present as a direct continuum of the extrapancreatic duct and have a luminal structure, as well as being devoid of enhanced GFP (EGFP) signal [although contained some H2BmCherry, which accumulates since histone 2B (H2B) stabilizes mCherry] (Fig. 1H). As these cells are positioned in between the extrapancreatic duct and intrapancreatic duct, we named them intermediate duct for ease of description. Conversely, the predefined Notch-responsive intrapancreatic ducts present long protrusions surrounding the principal islet and extending toward the pancreatic tail region that express higher mCherry signal. Notably, we also saw that a substantial number of the endocrine cells residing in the principal islet are weakly mCherry^+^, which is in line with the single-cell RNA-seq data showing the expression of *her15.1* in a subset of endocrine cells. Furthermore, we noted that a cytokeratin gene, *krt4*, is a marker gene of the large ductal cluster (Fig. 1C). Using HCR3.0 in situ hybridization, we found that extrapancreatic duct and intermediate duct are both *krt4*^+^. The intermediate ducts branch out to form a tree-like structure with a bifurcation toward the principal islet (Fig. 1I).

To better explore the pancreatic ductal system, we performed immunostaining in existing reporter lines or in situ hybridization in 6 dpf larvae for markers identified in the single-cell RNA-seq data from the adult zebrafish principal islet (i.e., not a direct validation due to different stages). The immunostaining of Vasnb in *Tg(-2.5cadherin17:mCherry)* [abbreviated as *Tg(cdh17:H2BmCherry)*] and *Tg(nkx2.2a:mEGFP)* suggested mosaic *cdh17* and *nkx2.2* expression patterns in the intermediate duct (fig. S2, A and B). We also observed *yap* activity and *pdx1* expression in both ducts and in a subset of the endocrine cells (fig. S2, C to E). In situ hybridization and immunofluorescent staining also demonstrated the expression of *id2a*, *sox9b*, *hnf1ba*, and *onecut1* in the intermediate duct (fig. S1, I to L). Notably, the ciliated cell master regulator, *foxj1a*, is restricted in the extrapancreatic duct as shown by both the transgenic reporter and the in situ hybridization (fig. S2F). Hence, the *krt4^+^* duct can be also molecularly classified into *foxj1a^+^* or *foxj1a*^−^ categories on top of the anatomical architecture. Together, we are able to visualize and molecularly define the whole ductal system in the zebrafish pancreas and distinguish novel ductal cell types, highlighting a ductal cell type showing specific *krt4* expression and differential Notch target gene activation pattern.

### Lineage tracing using canonical duct cell tracers only tracked a limited number of endocrine cells

**Fig. 2.**
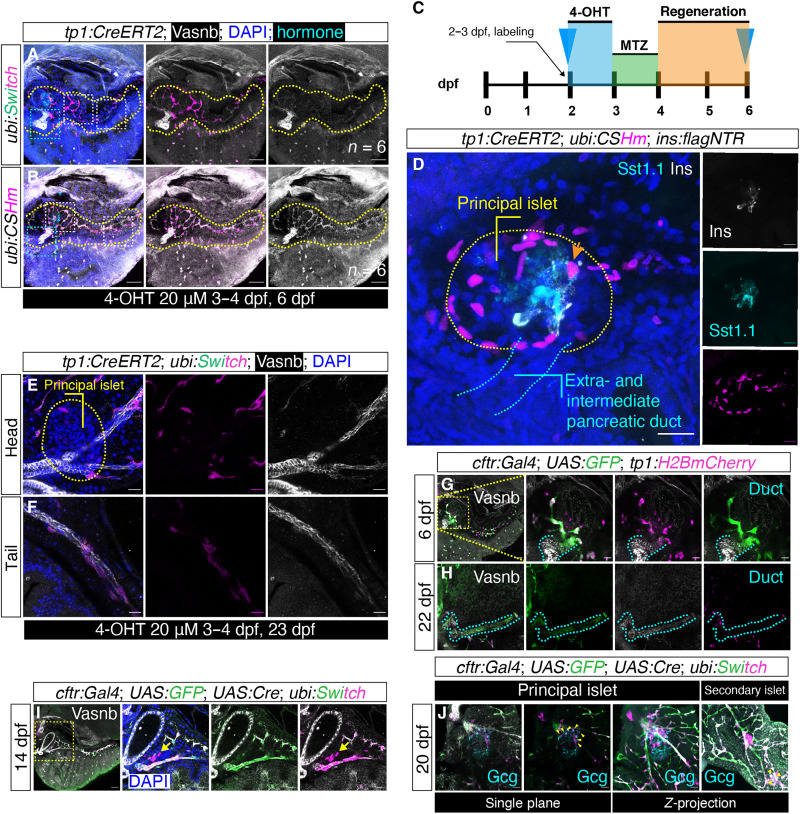
Lineage tracing showed limited Notch-responsive duct–to–endocrine cells conversion at the principal islet. (**A** and **B**) *Tg(tp1:CreERT2)* was crossed to two different color-switch lines under the control of *ubiquitin B* promoter. H2BmCherry/mCherry expression was specifically induced in *tp1^+^* Notch-responsive ducts and their progenies with 20 μM 4-OHT administered from 3 to 4 dpf, as visualized with *Z*-projections 6 dpf. Yellow, white, and cyan dashed lines indicate the pancreata, Notch-responsive intrapancreatic ducts, and non–Notch-responsive duct anterior to the principal islet, respectively. Scale bars, 100 μm. (**C** and **D**) Schematics and confocal *Z*-projections of *tp1* lineage–traced cells under β cell ablation condition. Tentative overlap in the *Z*-projection (the mCherry^+^ cell indicated by orange arrow) was confirmed to not coexpress Sst1.1 and insulin (Ins) in single planes. Scale bars, 40 μm. The yellow and blue dashed lines indicate the principal islet and extra- and intermediate pancreatic duct, respectively. (**E** and **F**) Long-term lineage tracing in *tp1:CreERT2;ubi:Switch* with 4-OHT treatment 3 to 4 dpf. Confocal *Z*-projections of pancreatic head (E) and tail (F) 23 dpf. Yellow dashed lines indicate the principal islet. Scale bars, 40 μm. (**G** and **H**) *cftr:Gal4;UAS:EGFP*;*tp1:H2BmCherry* indicate *cftr^+^* cells 6 dpf (G) and 22 dpf (H). The ductal cells are highlighted using cyan dashed lines. Scale bars, 40 μm. (**I** and **J**) *cftr:Gal4;UAS:EGFP;UAS:Cre;ubi:Switch* indicate *cftr^+^* lineage traced cells 14 dpf (I) and 20 dpf (J). Yellow dashed lines in (I) indicate the pancreatic head (magnifications in right panels). The yellow arrow points to lineage-traced endocrine cells. Yellow arrows in (J) indicate lineage-traced Gcg*^+^* cells in head and tail regions. Scale bars, 40 μm.

To address from which origin new postembryonic endocrine cells arise, we first used the *Tg(EPV.Tp1-Mmu.Hbb:Cre-ERT2,cryaa:mCherry)^s959^* [abbreviated as *Tg(tp1:CreERT2)*] line, which has been commonly used as a tracer of Notch-responsive cells and crossed it with two different responder lines: *Tg(ubi:loxp-EGFP-stop-loxp-mCherry)* [abbreviated as *Tg(ubi:Switch)*] and *Tg(ubi:loxp-CFP-stop-loxp-H2BmCherry)* [abbreviated as *Tg(ubi:CSHm)*]. We treated larvae with 4-hydroxytamoxifen (4-OHT) using maximum dosage (20 μM) at 2 or 3 dpf for 24 hours and analyzed the fate mapping results at 6 dpf. Apart from labeling the Notch-responsive intrapancreatic duct, neither extrapancreatic nor intermediate duct could be lineage traced or genetically labeled during development (Fig. 2, A and B). By using a nitroreductase/metronidazole (NTR/MTZ)–mediated cell ablation model (*ins:flagNTR*), we saw a similar result after β cell ablation with no lineage-traced insulin or somatostatin1.1 (Sst1.1)–positive cells, and the extrapancreatic and intermediate duct also appeared devoid of lineage tracing (Fig. 2, C and D). However, long-term lineage tracing (23 dpf) showed that a small proportion of the ductal cells located in the duct with a luminal structure could be genetically labeled (Fig. 2, E and F), in accordance with previous work and that we can see a small proportion of endocrine cells being labeled in a similar set up (fig. S10, B and D) (*31*). These results suggested that the *tp1* driven ductal tracer is useful in tracking the Notch-responsive cell lineage, whereas it is not qualified to trace the other ductal cells. It also prompts us to examine whether the previously uncharacterized ductal cell type might be a major source of endocrine cells in the pancreas.

To test our hypothesis, we used another duct tracer driven by the *cftr:Gal4* transgene. Staining for Vasnb in *TgBAC(cftr:Gal4);Tg(UAS:EGFP);Tg(tp1:H2BmCherry)* indicated a mosaic labeling pattern in the intermediate duct by the *cftr:Gal4* transgene (Fig. 2, G and H). Long-term lineage tracing experiments using *TgBAC (cftr:Gal4);Tg(UAS:EGFP);Tg(UAS:Cre);Tg(ubi:Switch)* juveniles demonstrated that a large number of intermediate duct can be genetically labeled, together with scattered fate-mapped endocrine cells sitting in proximity to the duct (Fig. 2, I and J). However, the mosaicism due to the nature of the *cftr* transgene and/or *Gal4/UAS* system may underestimate the number of new endocrine cells from the non–Notch-responsive duct, i.e., the intermediate duct. In sum, our lineage tracing results suggested that a surrogate source other than Notch-responsive duct serve as ductal progenitors with neogenic competence.

### Genetic fate mapping and targeted cell ablation using *krt4*^+^ knock-in iCre/CreERT2 lines

**Fig. 3.**
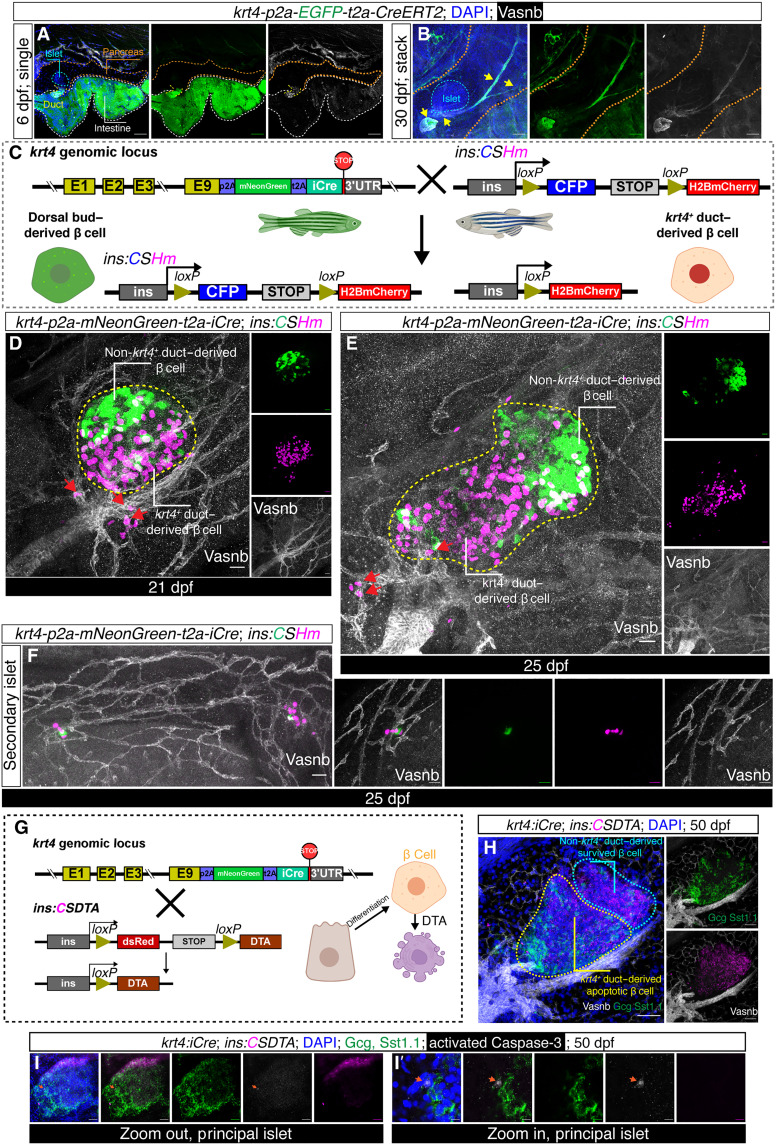
Lineage tracing and targeted cell ablation using *krt4* knock-in iCre line. (**A** and **B**) Confocal images of *krt4^+^* ductal cells in 6 dpf larvae (A) and 30 dpf juvenile fish (B). The brown, white, and cyan dashed lines indicate the pancreata, intestine, and the principal islet, respectively. The yellow arrows indicate large luminal *krt4^+^* ducts throughout the pancreas, i.e., even in the pancreatic tail as the fish grow older. Scale bars, 100 μm. (**C**) The *Cre/loxP* strategy used to demonstrate *krt4^+^* ductal cell–derived β cells. *krt4-p2a-mNeonGreen-t2a-iCre* was crossed to a color-switch line under the control of the insulin promoter. H2BmCherry is specifically induced in *krt4*-derived β cells, while β cells from other origins express CFP. (**D** and **E**) *Z*-projection showing differential distribution of β cells from *krt4*^−^ (green) and *krt4*^+^ (magenta) origins, 21 dpf (D) and 25 dpf (E). The ductal trees are visualized with anti-Vasnb staining. Scattered β cells budding out from the intermediate duct are highlighted with red arrows. White dashed lines indicate the principal islet. Scale bars, 40 μm. (**F**) Confocal images of *krt4*-derived β cells (magenta) in the secondary islets. Scale bars, 40 μm. (**G** to **I**) Design of targeted cell ablation of *krt4-*derived β cells by induction of DTA (G) with representative confocal images [(H) and (I)]. Cyan and yellow dashed lines in the *Z*-projection (H) indicate the area with surviving β cells at the posterior part of the principal islet and the β cell debris at the anterior part, respectively. The cell with orange arrow in (I) and magnified image (I′) is activated Caspase-3–positive, shown in single plane, indicating apoptosis. Scale bars, 80 μm.

Since the non–Notch-responsive ductal cells are *krt4*^+^ and to lineage trace these ducts and their descendants, we leveraged two recently published CRISPR-Cas9 3′ knock-in tracers generated in our laboratory, i.e., *TgKI(krt4-p2a-mNeonGreen-t2a-iCre)* [abbreviated as *TgKI(krt4:iCre)*] and *TgKI(krt4-p2a-EGFP-t2a-CreERT2)* [abbreviated as *TgKI(krt4:CreERT2)*] (*47*). The different Cre lines complement each other in that the *iCre* line gives high recombination efficiency but may have minor leakage, whereas the *CreERT2* line enables tight control but with lower recombination efficiency. These knock-in lines have been shown to recapitulate *krt4* expression in the skin and intestine, and we also confirmed the fluorescent signals in the pancreatic ducts with luminal structure, specifically in the extrapancreatic duct in 6 dpf larvae and later also in all large luminal ducts throughout the pancreatic tail (Fig. 3, A and B). For the lineage tracing experiment, we first crossed the *TgKI(krt4:iCre)* with the *Tg(ins:loxP-CFP-stop-loxP-H2BmCherry)^KI141^* [abbreviated as *Tg(ins:CSHm)*] responder (Fig. 3C). The rationale for this is that, in the *krt4-*expressing ductal cells, the genetic cassettes in between two *loxP* sites will be excised and only the lineage-traced β cells would have H2BmCherry expressed in the nucleus. Therefore, the first phase of β cell differentiation derived from the dorsal bud and other *krt4^−^* sources would retain the cyan fluorescent protein (CFP) expression, whereas the β cells derived from the *krt4^+^* duct would be H2BmCherry^+^. We observed that the CFP-labeled old β cells are mostly located at the posterior region of the principal islet (Fig. 3, D and E), in line with previous findings (*30*). In contrast, the *krt4*-derived β cells (which in 3-to-4-week-old larva made up >70% of all β cells) are mostly located anterior to the β cells derived from a *krt4*^−^ origin (table S1). With staining for Vasnb, we could readily visualize that the *krt4*-derived β cells (magenta) are budding out from the intermediate duct (Fig. 3, D and E). Moreover, we saw that β cells within the secondary islets are mostly H2BmCherry^+^, although intermingled with a few scattered CFP^+^ β cells (indicative of Notch-responsive duct–derived β cells) (Fig. 3F).

To validate the above findings, we designed a complementary targeted cell ablation approach using a transgenic line expressing an inducible diphteria toxin chain alpha (DTA) suicide transgene under the control of *ins* promoter (Fig. 3G). The rationale is that if *krt4^+^* ducts serve as the progenitor cells, then their β cell descendants would be specifically ablated, while β cells of other origins would stay intact. Given that the ablation efficiency is highly dependent on the activity of *ins* promoter, we applied this system only in the basal state, as the *ins* promoter activity will diminish during regeneration and thereby not be sufficient to induce effective ablation. We crossed the *TgKI(krt4:iCre)* with *Tg(ins:loxp-dsRed-stop-loxp-DTA)* and costained the juvenile pancreata with glucagon and Sst1.1 antibodies to depict the principal islet. The β cells in the anterior regions of the principal islet were apoptotic, as displayed by the scattered dsRed debris, while a small number of β cells (indicative by intact dsRed signal) at the posterior part of principal islet were retained (Fig. 3H). Moreover, immunostaining of activated Caspase-3 indicated scattered apoptotic cells lining the border of the principal islet (Fig. 3I).

**Fig. 4.**
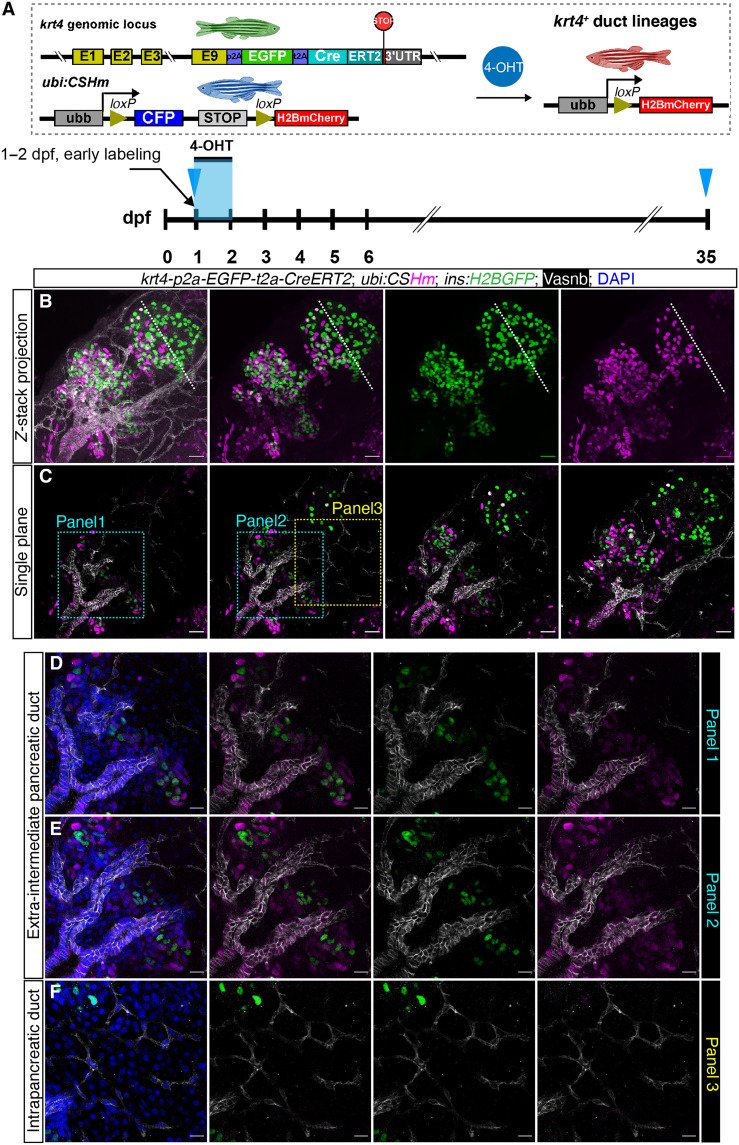
Spatiotemporal-controlled lineage tracing of *krt4^+^* ducts. (**A**) Workflow of the *Cre/loxP* system used for spatiotemporal lineage tracing of *krt4^+^* ductal cells. Fish carrying *krt4-p2a-EGFP-t2a-CreERT2* (identified by green skin) are crossed to fish carrying *ubi:CSHm* transgenic lines (identified by blue skin). The 4-OHT is administered from 1 to 2 dpf, and we analyzed 35 dpf juvenile fish. (**B**) The maximum *Z*-projection of confocal images showing the islets from *TgKI(krt4:CreERT2);Tg(ins:H2BGFP);Tg(ins:CSHm)* line stained with Vasnb antibody (white) and 4′,6-diamidino-2-phenylindole (DAPI). All β cells are displayed with green fluorescence (from the *ins:H2BGFP* transgene) in the nucleus, and *krt4-*derived endocrine and ductal cells that can be traced back to *krt4^+^* duct origin expressed H2BmCherry. Notably, the luminal duct in the pancreatic tail and Notch-responsive ductal cells are devoid of H2BmCherry. The fluorescence in *krt4*^+^ ducts is not visible because of its low intensity and the used confocal microscopy setting. (**C** to **F**) Single-plane confocal images showing the labeling pattern in three different layers/regions, with the cyan and yellow outlined regions (C) magnified in (D) to (F). Scale bars, 80 μm [(B) and (C)] and 40 μm [(D) to (F)].

For further confirmation and to avoid possible false conclusions based on potential leakage in the *iCre* line, we temporally labeled the *krt4^+^* cells in *TgKI(krt4:CreERT2)*;*Tg(ubi:CSHm)* with 4-OHT from 1 to 2 dpf (Fig. 4A and fig. S3, A and B). Immunostaining of 35 dpf juveniles showed that the *krt4*-traced H2BmCherry^+^ β cells are predominantly located at the anterior region of the principal islet (Fig. 4B). Moreover, we saw extrapancreatic and intermediate duct H2BmCherry labeling, while the fluorescent signals disappeared distal to the ductal bifurcation region. This suggests that the luminal ducts along the tail of the pancreas, despite forming a direct continuum with the intermediate duct around the principal islet, originate from a source other than the intermediate duct (Fig. 4, C to F).

Unlike mammals, *neurog3* does not function as a pioneering factor for β cell neogenesis in zebrafish. Instead, previous findings showed that two other basic helix-loop-helix transcription factors, *ascl1b* and *neurod1*, play combinatorial roles as endocrine progenitors in zebrafish (*48*). Thereby, we assumed that the real ductal progenitor cells will up-regulate the expression of the above two transcription factors in the context requiring accelerated neogenesis, i.e., in the chemogenetic β cell ablation model [using *Tg(ins:FLAG-NTR,cryaa:mCherry)^s950^*, abbreviated as *Tg(ins:flagNTR)*]. We observed that upon β cell ablation, there are a substantial number of *ascl1b:EGFP*^+^ cells either positioned in the intermediate duct region or budding out from the ductal tree, a process sustained at least until 1 day postablation (dpa) in larvae (fig. S4, A and B, and tables S2 and S3). The immunostaining results using the *neurod1:EGFP* reporter showed similar results, consistent with the prior time-lapse imaging study indicating the presence of endocrine cells in/along the duct anterior to the principal islet during early development (fig. S4, C and D) (*49*).

In older larvae, to our surprise, we noted that even without β cell ablation, a large number of cells in the duct with luminal structure are *ascl1b:EGFP^+^* and *tp1:H2BmCherry^−^*, indicating that those luminal ductal cells with lower Notch activity might be primed for differentiation (fig. S4E). We also found that the expression of *ascl1b* is restricted to the ductal cells (mainly intermediate duct) upon β cell ablation (fig. S4F). Immunostaining using *neurod1:EGFP* transgenics in the basal state showed that large numbers of EGFP^+^ cells are located at both the proximal and distal luminal ducts (fig. S4, G and H). *Z*-stack movies showed the neogenic endocrine cells (indicated by *neurod1:EGFP*) residing in, along, and closely attaching to the duct (movies S1 to S3). Nevertheless, when treated with low dosage of Notch inhibitor (LY411575, 1 μM), *ascl1b:EGFP* was markedly expressed only in the *tp1*-driven Notch-responsive duct, indicating that the naturally occurring neogenic program differs from the pharmacological Notch inhibition and that different ductal cell types engage in the processes (fig. S4, I and J). Together, our results provide concrete evidence demonstrating that the *krt4^+^* ducts are progenitors giving rise to the majority of postembryonic endocrine cells.

### Lineage tracing β cell regeneration

To further interrogate the regenerative framework of β cell neogenesis in zebrafish, we specifically ablate β cells from 3 to 4 dpf and allow β cell regeneration for additional 2 days. To better quantify the contribution of *krt4^+^* duct to β cell regeneration, we switched to *Tg(ubi:CSHm)* and counted the number of traced insulin-producing cells in the principal islet (fig. S5A). We observed that around 40% insulin-producing cells in the principal islet can be lineage-traced and are located in the region close to the *krt4*^+^ ductal bifurcation (fig. S5, B to E, and tables S4 and S5). Notably, these cells also express *sst1.1*, demonstrating a bihormonal state. However, the other half of regenerated insulin-producing cells are distributed mainly at the distal region of principal islet and are also *sst1.1*^+^. The long-term lineage tracing experiment showed that most insulin– and Sst1.1–double positive cells turn into monohormonal cells (>95%), at least when the β cells were ablated at the larval stage, implicating an adaptation to the basal physiological states in developing/growing zebrafish (fig. S5F and table S6). Last, constitutive and temporal lineage tracing experiments also indicated that two other major endocrine cell types in the islet, Gcg^+^ and Sst1.1^+^ cells, can also be derived from the *krt4^+^* duct origin (fig. S5, G and H).

To have a comprehensive understanding of different cellular behaviors during insulin-producing cell recovery, we leveraged multiple endocrine cell tracers for lineage tracing experiments during β cell regeneration. We first applied a pan-endocrine tracer: *Tg(neurod1:CreERT2, cryaa:Venus)^KI137^* [abbreviated as *Tg(neurod1:CreERT2)*] with temporal labeling before β cell ablation. Although the *neurod1* tracer resulted in mosaic labeling of the endocrine cells, there is no ectopic labeling (leakage) in the ductal cells (fig. S3C). We found that *neurod1-*derived insulin–producing cells are dispersedly distributed in the principal islet after 2 days of regeneration in both 6 dpf larvae and 31 dpf juveniles (fig. S6, A to G). These cells are all insulin– and Sst1.1–double positive. Further, to rule out the cell fate conversion from δ (*sst2^+^*), α, and *gip^+^* cells, we performed fate mapping using *Tg(sst2:Cre; cryaa:YFP)^s963^* [abbreviated as *Tg(sst2:Cre)*], *TgBAC(arxa:Cre)*, and *Tg(gip:iCre)*, respectively, and only observed a very limited number of insulin-producing cells that could be traced back to their origins (fig. S7, A to I, and tables S7 to S9). Furthermore, we did not see significant changes of lineage-traced insulin-expressing cells, indicating that these three cell types were not highly responsive to injury and do not initiate cellular conversion upon β cell ablation (fig. S7, J to L, and tables S10 to S12). In sum, these data support the previous findings regarding *sst1.1^+^* endocrine cells converting to insulin-producing cells and highlight a cellular plasticity in multiple cell types, which might account for the exceptional regenerative capacity of β cells in zebrafish.

### Concurrent ductal remodeling and transcriptional changes

**Fig. 5.**
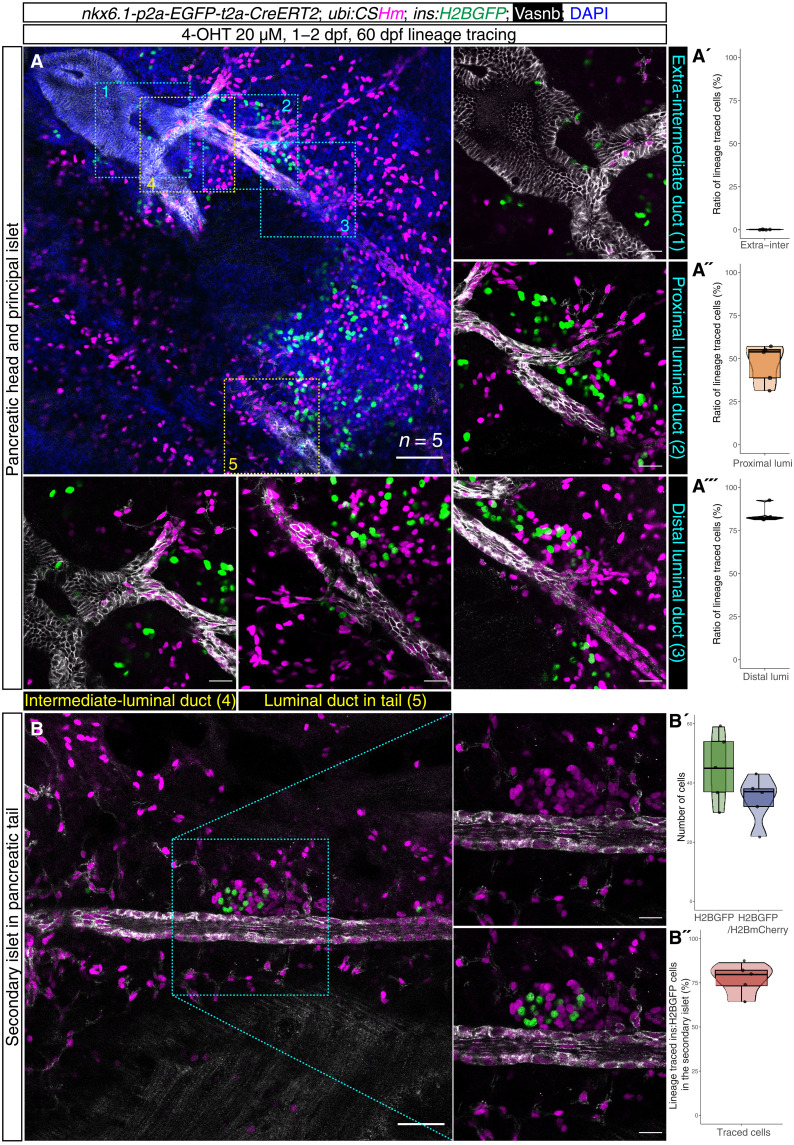
Spatiotemporal-controlled lineage tracing of *nkx6.1^+^* ducts. (**A**) Single-plane confocal images showing the labeling pattern in *TgKI(nkx6.1:CreERT2);Tg(ubi:CSHm)* treated with 4-OHT from 1 to 2 dpf, displaying five distinct regions with high magnifications shown on the right and bottom, respectively. The extrapancreatic and intermediate ducts are devoid of labeling, while the luminal ducts in the pancreatic tail and Notch-responsive intrapancreatic ducts can be traced back to the *nkx6.1*^+^ cell origin. The ductal cells residing in between the intermediate duct and the luminal duct in the tail regions are devoid of labeling. The quantification results of the proportion of lineage traced ductal cells are displayed in (A′) (extra-intermediate duct region), (A″) (proximal luminal duct), and (A‴) (distal luminal duct). (**B**) Single-plane confocal images showing luminal duct and lineage-traced β cells in a secondary islet. The luminal ducts in the pancreatic tails are H2BmCherry^+^. The coexpression of H2BGFP and H2BmCherry indicate β cells within the secondary islet that can be traced back to the *nkx6.1^+^* cell origin. The quantification results of *ins:H2BGFP*–single positive cells and *ins:H2BGFP/H2BmCherry*–double positive cells are shown in (B′), with the proportion of lineage traced *ins:H2BGFP*^+^ cell shown in (B″). Five size-matched secondary islets were used for the quantification. Anti-Vasnb (white) and *ins:H2BGFP* transgene (green) were used to visualize the ductal tree and the islets. Scale bars, 80 μm.

As we showed that the *krt4^+^* duct in the pancreatic tail does not originate from the *krt4^+^* extrapancreatic and intermediate duct (Fig. 4, B to F), our next question was where and how the *krt4^+^* ducts in different regions are formed and assembled to build a continuous organized luminal ductal system. We used our recently generated *nkx6.1* knock-in CreERT2 line [*TgKI(nkx6.1-p2A-EGFP-t2A-CreERT2)^KI132^*, abbreviated as *TgKI(nkx6.1:CreERT2)*] and *tp1:CreERT2* transgenics to lineage trace their descendants from 1 to 2 dpf (for *nkx6.1*) and 2 to 3 dpf (for *tp1*), respectively. For *TgKI(nkx6.1:CreERT2)*, the administration of 4-OHT achieves maximum labeling efficiency in the Notch-responsive intrapancreatic duct, while the labeling in the extrapancreatic and intermediate duct has high variability from almost no labeling to >50% labeling (fig. S8, A to C, and table S13). We took advantage of this varied labeling pattern in the larval stage to better delineate the allocation of ducts in different regions of the adults. The long-term lineage tracing results showed that the luminal duct located in the pancreatic head and tail regions is mostly mCherry^+^ with a large number of lineage-traced β cells attached to it, while there is no lineage-traced β cells sitting close to the Notch-responsive duct (Fig. 5A and tables S14 to S16). The luminal ductal cells in the junction region, i.e., the intermediate duct anterior to the principal islet, are mostly mCherry^−^ in half of the samples, while the other half of the samples can obtain mCherry labeling in extrapancreatic and intermediate ducts (fig. S8). These results indicated that the Notch-responsive intrapancreatic duct might undergo a marked transformation and gradually assemble to form a luminal tubular structure in the pancreatic tail (Fig. 5B and tables S17 and S18).

For further validation, we performed a long-term lineage tracing experiment using *tp1* tracer restricted to the Notch-responsive duct. We confirmed that the *tp1-*driven ductal line can barely label extrapancreatic and intermediate ducts in the larval stage (fig. S8, D and E). We noted that the luminal duct in the tail region shows mosaic labeling, while there is an absence of mCherry signal in the intermediate ductal cells and in the extrapancreatic duct at the early juvenile stage (fig. S10). Moreover, we also confirmed that the *tp1*-traced descendant cells in the luminal ducts overlap with *krt4*, further confirming the remodeling process (fig. S11). Last, immunostaining of the *foxj1a:EGFP* reporter and *Tg(foxj1a:iCre);Tg(ubi:CSHm)*-traced juveniles showed that the *foxj1a^+^* cells and their descendants only appeared in the extrapancreatic duct, demonstrating that there might be at least three different duct origins (*krt4^+^/foxj1a^+^*, *krt4^+^/foxj1a^−^*, and *krt4^−^/tp1^+^*) that develop independently and coordinate with each other to build an intricate *krt4^+^* luminal ductal system with cells of similar morphology, transcriptional signatures, and neogenic competence (fig. S12).

### Single-cell transcriptomics specifies a differentiation trajectory and endocrine progenitor cells

To further explore the cellular and molecular events underlying duct-to-endocrine cell neogenesis, we performed single-cell RNA-seq of adult zebrafish pancreata (6 to 9 months old) isolated from *TgKI(krt4:iCre);Tg(ubi:CSHm);Tg(ins:flagNTR)* adult fish in the basal state and after β cell ablation. We performed fluorescence-activated cell sorting (FACS) to sort out mCherry^+^/DAPI^−^ cells, followed by droplet-based sequencing using the 10× platform (fig. S13A). To elucidate the complexity of cellular behavior in both the acute and sustained phase of regeneration, we selected 2, 5, or10 days after the first dose of MTZ (presented as 2, 5, or 10 dpa, single ablation) and 2 or 5 days after the second dose of MTZ (presented as 2 or 5 dpa, double ablation). We left 1 month interval between the first and second MTZ dose because within this time, the adult fish could accomplish full recovery of hyperglycemia and the replenished insulin-producing cells had up-regulated *ins* promoter transcriptional activity (i.e., to enable an efficient second ablation) (fig. S13, B and C).

**Fig. 6.**
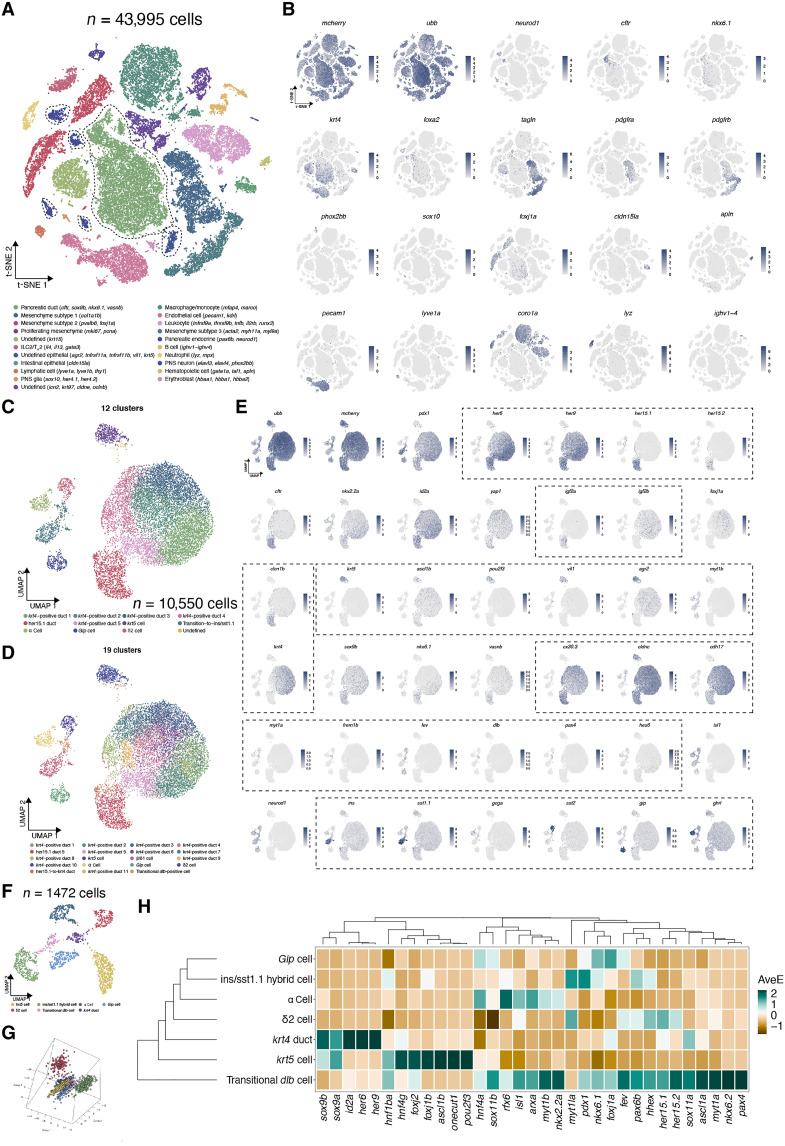
Single-cell transcriptomics highlight distinct molecular signatures in various cell types. (**A**) t-distributed stochastic neighbor embedding (t-SNE) plot showing the cell type assignment of all single cells (~40,000). The classifications were based on previously known marker genes that were significantly enriched in each cluster (highlighted in parentheses). T_H_2, T helper 2 cells; ILC2, innate lymphoid cells type 2. (**B**) t-SNE plots of well annotated cell markers, colored by the normalized gene expression levels. (**C** and **D**) UMAP plot of subclustering including pancreatic duct, *krt5^+^* cells, and endocrine cells (~10,000). (**E**) UMAP plots of various marker genes, colored by the normalized gene expression levels. (**F** and **G**) UMAP plot and 3D PCA plot showing the embedding of *krt5^+^* cell and all endocrine cells. (**H**) Heatmap highlighting the key lineage-committed transcription factors and hormones in each cluster, with colors displayed on column-scaled mean expression. The rows and clusters were ordered by hierarchical clustering of scaled expression values.

We sequenced >40,000 cells, and after regular quality control, cell filtering, dimension reduction, and graph-based clustering, we identified 21 clusters in total (Fig. 6A). We applied well-defined cell markers based on prior knowledge and identified endocrine cells (*neurod1*), pancreatic duct (*cftr*, *krt4*, *cdh17*, and *vasnb*), stromal cells (*tagln* and *acta2*), smooth muscle cells and pericytes (*pdgfra* and *pdgfrb*), endothelial cells and lymphatic cells (*pecam1* and *lyve1a*), leukocytes (*ptprc* and *coro1a*), erythrocytes/red blood cells (*hbaa1*), B cells (*ighv1 to ighv4*), peripheral nervous system neurons (*elavl3* and *elavl4*), peripheral nervous system glia (*sox10*), and innate lymphoid cells type 2 (ILC2)/T helper cells (T_H_2) (*runx3* and *il4*) (Fig. 6B; https://olovanderssonlab.shinyapps.io/neogenesis/). We also defined one small cluster as intestinal epithelial cell contamination due to very specific expression of the tight junction gene *cldn15la* (*50*). Moreover, we identified a small cluster with specific expression of *krt5* and many other epithelial cells markers. We also included this cluster in the downstream analyses as we ruled out the possibility of skin cell (*tp63* and *krtt1c19e*) and gallbladder cell (*sstr5*) contamination (*51*). To extrapolate the detailed cell embeddings of pancreatic duct and endocrine cells, we manually subselected cells belonging to the endocrine, pancreatic duct, and *krt5^+^* cell clusters (~10,000 cells) and re-ran the analytical pipeline (Fig. 6, C and D).

The pancreatic ductal cells can be further categorized into two major clusters, with the majority showing high expression of *krt4*, *her6*, and *her9* (*her6/9* are zebrafish homologs of *HES1/4*), indicating *krt4^+^* luminal ductal cells. The smaller ductal cluster is defined as Notch-responsive duct because it displayed preferential *her15.1* and *her15.2* (zebrafish homologs of *HES5*) expression (Fig. 6E). Such a molecular signature is generally in line with the previous single-cell RNA-seq dataset. In addition, we noted that *igf2a* and *igf2b* are marker genes for these two types of ducts, respectively. The *krt5^+^* cell cluster presents a distinct expression pattern indicated by several transcription factors, including *ascl1b*, *myt1b*, and *pou2f3* (Fig. 6E). Further, these *krt5^+^* cells harbor many molecular signature genes that have been described as markers of goblet cells and paneth cells in the intestine (including *agr2*, *spry2*, *galnt7*, *tnfrsf11a*, and *sox8b*) (*52*), indicating functional similarities. The endocrine clusters can be subdivided into four major categories using low resolution (0.2), i.e., *ins*/*sst1.1* hybrid cells, *gcga*/*ghrl* cells, δ cells (*sst2^+^*), and *gip* cells. We managed to obtain a more delicate clustering using higher resolution (0.7) and identified a new cluster that was previously part of the *ins*/*sst1.1* hybrid cells (Fig. 6D). These cells were featured by specific expression of the Notch ligand, *dlb* (the orthologs of *Dll1/DLL1*), which could mediate lateral inhibition in a differentiating endocrine progenitor (*53*, *54*). Moreover, these cells up-regulated the Notch negative regulator *hes6*, suggesting diminished Notch activity. To better display the lineage path of *dlb^+^* cells to *ins/sst1.1* hybrid cells without becoming skewed from a large number of pancreatic ductal cells, we performed one more subclustering to exclude *krt4^+^* duct and Notch-responsive duct clusters (Fig. 6F). We identified eight clusters, with some remaining *krt4^+^* ductal cells positioned in the center and *dlb*^+^ cells positioned in between *krt4^+^* duct and *ins/sst1.1* hybrid cells of the Uniform Manifold Approximation and Projection (UMAP) embedding. Likewise, the three-dimensional (3D) principal components analysis (PCA) plot also showed a trajectory from *krt4^+^* duct to *dlb^+^* cells to *ins/sst1.1* hybrid cells in the 3D space (Fig. 6G). Notably, the heatmap of canonical transcription factors in pancreatic cell fate commitment and pancreatic hormones showed the up-regulation of many endocrine-related genes in *dlb^+^* cells, including *pax4*, *pax6b*, *isl1*, *myt1a*, *sox11a*, *sox11b*, and *fev* (Fig. 6H). Considering that these cells are multihormonal, as well as *dlb*^+^ and *hes6*^+^, we propose that they stand for a novel and rare transitional cell population with endocrine precursor (EP) properties.

### Pseudotime and regulon reveal gene regulatory networks along the differentiation trajectory

**Fig. 7.**
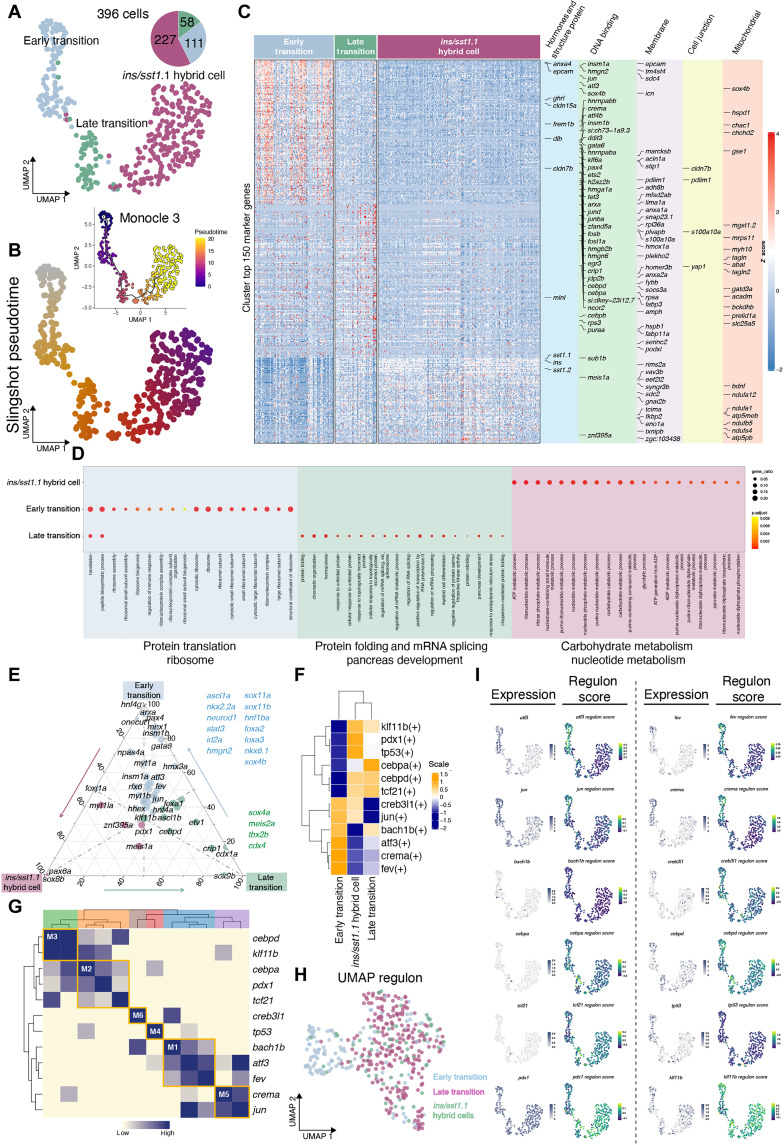
In silico analyses of transition-to-*ins^+^/sst1.1^+^* hybrid cells. (**A**) UMAP plot displaying three clusters of transition-to-*ins^+^/sst1.1^+^* hybrid cells, color-coded by subpopulation. The numbers and proportions of each cell type were displayed in a pie chart. (**B**) The Slingshot and Monocle 3 analyses reveal a linear trajectory, with early transition cells predominantly early in pseudotime (left), late transition cells in the middle, and *ins^+^/sst^+^* hybrid cells predominantly later in pseudotime (right). (**C**) Heatmap of the top 150 differentially expressed genes per cluster, with cell markers gene, DNA binding genes, tight junction genes, membrane protein gene, and mitochondrial genes highlighted on the right. (**D**) Gene Ontology (GO) analysis of differential expressed genes in each cell cluster along the transition-to-*ins^+^/sst1.1^+^* hybrid cells. Dot plots showing the differentially enriched GO terms colored by adjusted *P* value and sized by gene ratio. (**E**) Ternary plot shows the expression of DNA binding genes in early transition, late transition, and *ins^+^/sst1.1^+^* hybrid cells. Genes with highest percentage of expression in early transition cells are in blue, Late transition in green, and *ins^+^/sst1.1^+^* hybrid cells in red. (**F**) Heatmaps showing the enriched regulons among the three cell states in the trajectory. The regulons were ordered by hierarchical clustering. (**G**) Heatmap showing the hierarchical clustering of identified regulon and four regulon modules. (**H**) The UMAP for transition-to-*ins^+^/sst^+^* hybrid cells based on the regulon activity scores (RASs), each cell is color-coded on the basis of the cell states assignment. (**I**) UMAP projection of transition-to-*ins^+^/sst1.1^+^* hybrid cells overlayed by the expression level of transcription factors (left) and RAS (right).

To unravel the molecular events underpinning the cell state transition between *dlb^+^* cells and *ins/sst1.1* hybrid cells, we first subset these cells and performed pseudotime analyses using different algorithms (slingshot and Monocle 3). We identified a unified lineage path starting from the *dlb*^+^ cells toward *ins*/*sst1.1* hybrid cells. To better uncover the distinct molecular pathways enriched in different cells, we set a new resolution as 0.3 and redefined the cells within the trajectory into early transition, late transition, and ins/sst1.1 hybrid cells (Fig. 7, A and B). Differential gene expression analyses among these three clusters highlighted a large number lineage-committed transcription factors (*insm1a*, *atf3*, *sox4b*, *insm1b*, *gata6*, *klf6b*, *pax4*, *arxa*, and *ets2*), injury responsive transcription factors (*jun*, *jund*, *junba*, *fosb*, *foxl1a*, *cebpd*, and *cebpa*) (*55*, *56*), epigenetic regulators (*tet3*), and other DNA binding/RNA splicing proteins (*hmga1a*, *hmgb2b*, *hmgn6*, and *hmgn2*) that are highly expressed in the early transition cells (Fig. 7C). Furthermore, the early transition cells also retain the expression of various cell membrane/junctional proteins that are indicative of epithelial cell properties (*cldn7b*, *anxa4*, *epcam*, *tm4sf4*, and *cldn15a*). These genes gradually decline when cells transit toward *ins/sst1.1* hybrid cells. Gene enrichment analyses revealed that genes involved in protein translation and ribosomal biogenesis are highly expressed in the early transition cells, while the up-regulated genes in the late transition cells are engaged in protein folding, mRNA splicing, and pancreas development (Fig. 7D). The *ins*/*sst1.1* hybrid cells are enriched in genes related to carbohydrate and nucleotide metabolism. When we ordered the DNA binding proteins and lineage-committed transcription factors based on the expression levels (regardless of whether they are differentially expressed), we found that most of them are relatively highly expressed in the early and late transition and are down-regulated when cells become *ins*/*sst1.1* hybrid cells (Fig. 7E).

These results further prompted us to perform regulon analyses to find the key transcriptional regulatory networks leading to the cell state transition. We found 12 regulons, which could be clustered into six modules, with crema, atf3, creb3l1, fev, bach1b, and jun regulons enriched in the early transition cells, cebpa, cebpd, and tcf21 regulons featured in the late transition cells, and pdx1, klf11b and tp53 regulons, are mostly functional in the *ins*/*sst1.1* hybrid cells (Fig. 7, F and G, and fig. S14). In addition, we used the regulon score to recluster the transition-to-*ins/sst1.1* hybrid cell lineage and noted that early transition cells were very well separated from the other cells, whereas the other two clusters intermingled with each other, suggesting that the transcriptional state of the late transition cells is more similar to that of the *ins*/*sst1.1* hybrid cells (Fig. 7H). We also mapped the gene expression level and the regulon score in parallel on UMAP and obtained consolidated evidence of the leading regulons, i.e., atf3, fev, jun, and crema for early transition cells, cebpd for late transition cells, as well as pdx1 and klf11b in *ins/sst1.1* hybrid cells (Fig. 7I).

### Velocity-based methods identify dedifferentiation and differentiation events

**Fig. 8.**
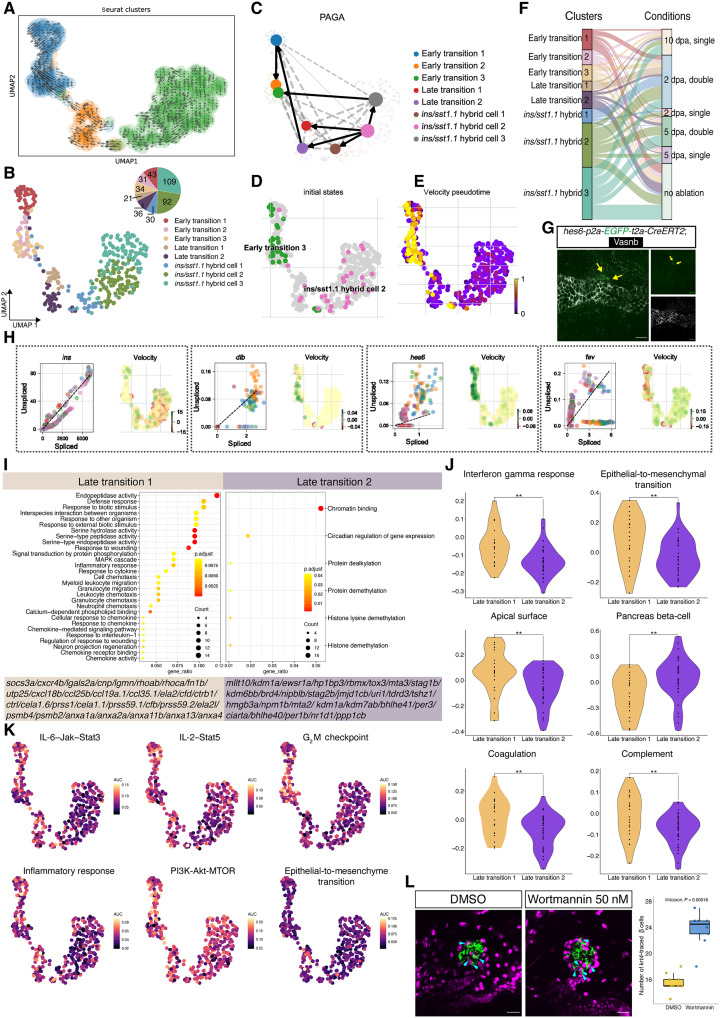
Velocity-based analyses and pathway validation. (**A**) UMAP colored by latent time progression. Cells later in the trajectory (determined by RNA velocity) are yellow. The UMAP is overlaid by arrows indicating the extrapolated future cell states identified through RNA velocity analysis, suggesting that both differentiation and dedifferentiation processes occur simultaneously during insulin-producing cell recovery. (**B**) UMAP projection of transition-to-*ins^+^/sst1.1^+^* hybrid cells with eight subclusters (three early transition cell states, two late transition cell states, and three *ins^+^/sst1.1^+^* hybrid cell states). (**C**) The PAGA analysis indicates dedifferentiation process. (**D** and **E**) The root identification and velocity-based pseudotime results by CellRank. (**F**) Graphical summary using Sankey plots demonstrating the relationship between cell states (left) and condition (right). (**G**) Representative single-plane confocal image of *hes6*^+^ cells from the immunostaining of the knock-in *hes6-p2a-EGFP-t2a-CreERT* zebrafish line. The yellow arrows point to two EGFP^+^ cells adjacent to the duct. (**H**) Scatterplots showing the level of unspliced versus spliced transcripts of cell state specific genes. (**I**) GO analysis of up-regulated genes expressed in late transition 1 and late transition 2 cells, with enriched gene shown below. Dot plots showing the differentially enriched GO terms colored by adjusted *P* value and sized by gene ratio. MAPK, mitogen-activated protein kinase. (**J**) Violin plots and dot plots highlighting the differential gene set scores between late transition 1 and late transition 2 cells using single-cell gene set variation analysis. (**K**) UMAP plot showing the statistically significant gene activity scores in each cell. IL-6, interleukin-6; Jak, Janus kinase; Stat3, signal transducers and activators of transcription 3; AUC, area under the curve. (**L**) Representative single-plane confocal images and quantifications of β cells following treatment with either dimethyl sulfoxide (DMSO) or the PI3K inhibitor wortmannin. Treatment with wortmannin increased the number of newly generated *krt4*-derived β cells (mCherry^+^/insulin^+^; Wilcoxon test) in the basal state (without β cell ablation). Scale bars, 40 μm.

Next, to further dissect the lineage path of the transition-to-*ins/sst1.1* hybrid cells, we performed multiple analyses based on the mRNA splicing information. Velocity analyses showed that most cells have velocity vectors pointing toward the early transition cell state. We identified two distinct vectors in the late transition cluster, with a subset of cells in the upper cluster showing fate direction toward the *ins*/*sst1.1* hybrid cells, while the lower cluster was going backward (Fig. 8A). To distinguish the varied cellular behavior, we proceeded with further subclustering using high resolution (0.7) and identified eight clusters, named early transitions 1, 2, and 3; late transitions 1 and 2; *ins*/*sst1.1* hybrid cells 1, 2, and 3 (Fig. 8B). In addition, the PAGA algorithm identified that *ins/sst1.1* hybrid cell 2 and early transition 1 cells serve as the major root of dedifferentiation and differentiation, respectively (Fig. 8C). Consistently, the CellRank algorithm also determined two roots located at early transition cells and *ins*/*sst1.1* hybrid cells, respectively (Fig. 8, D and E).

We also found that cells isolated from the 2 dpa double ablation condition contributed to the majority of cells in early and late transition states (Fig. 8F). Cells in the transitional states up-regulate *dlb* and *hes6.* For biological validation, we generated a knock-in at the *hes6* locus *TgKI(hes6-p2a-EGFP-t2a-CreERT2)*, visualizing its expression. Immunostaining of juvenile fish revealed *hes6*^+^ cells residing just adjacent to the luminal duct (Fig. 8G), a position consistent with the expected nature of the transitional cells. The scatterplot revealed that the late transition 1 cells have a higher number of cells with more unspliced insulin mRNA versus the spliced form, suggesting that these cells are undergoing differentiation toward β cells, while the late transition 2 cells show the opposite pattern (Fig. 8H). Cells in early transition showed more abundant unspliced *dlb* and *hes6* gene expression, indicating a progenitor status, while cells in the *ins/sst1.1* hybrid cells demonstrated higher amounts of unspliced *fev*, indicating a precursor status (Fig. 8H). Notably, we found that the late transition 1 cells mostly originate from the 2 dpa double ablation group, indicating that the differentiation process is mainly initiated upon the acute phase in the double ablation condition (Fig. 8H).

Differential gene expression between the two late transition clusters revealed that late transition 1 cells are characterized by the up-regulation of a variety of immune-related genes (Fig. 8I). Contrarily, the late transition 2 cells showed elevated expression of epigenetic regulators and chromatin remodelers (Fig. 8I). Single-cell gene set variation analyses and pathway activity analyses further demonstrated that the late transition 1 cells contain molecular signatures of inflammation, epithelial-to-mesenchymal transition (EMT), and an apical surface, while late transition 2 cells have a higher molecular signature of genes for pancreas development (Fig. 8J). This indicated that the late transition 1 cells are more similar to the progenitor state and the late transition 2 cells preserve more molecular properties of endocrine cells (Fig. 8J). Further, we interrogated the activity scores of the hallmark gene sets and showed that the PI3K-Akt-mTOR and gene sets involved in inflammatory response activities are highest in early transition and gradually declined along the trajectory (Fig. 8K). On the basis of a bimodal model, we set 0.15 as the threshold for activated PI3K-Akt-mTOR signaling and find that >40% (42.3 and 41.4%) cells in early or late transition cells are PI3K-Akt-mTOR^high^, while the percentage was 27% cells in insulin^+^/Sst1.1^+^ hybrid cells. Considering that insulin signaling is involved in the activation of the PI3K-Akt-mTOR pathway, we assumed that the general inhibition of PI3K-Akt-mTOR might promote the transition-to-*ins*/*sst1.1*. We tested our hypothesis by treating *krt4* lineage–traced larvae with the broad PI3K inhibitor wortmannin and observed an increased number of *krt4* lineage–traced insulin^+^ cells (Fig. 8L and table S35). These data correlate with previous findings (*57*) and serve as proof of concept for how induction of β cell neogenesis targets can be derived from a comprehensive single cell transcriptomics analysis.

### Cellular conversion shares similarities in transcriptomic changes with neogenesis

Since the insulin-producing cells we captured were mostly multihormonal, we leveraged a recently published single-cell dataset that was specifically enriched for β cells with or without ablation (fig. S15) (*37*). Aiming to reveal the different transcriptional profiles between mature β cells and regenerative insulin–producing cells, we filtered out other cell types (including multihormonal cells) and only kept the β cells and *ins/sst1.1* hybrid cells. We identified a UMAP embedding with cells demonstrating a sequential course from early regeneration stage to late regeneration stage to mature β cells (fig. S15, A to D). We manually defined them into five clusters, named Hybrid 1/2/3 and Beta 1/2 cells. The Hybrid 1 cells are mostly composed of hybrid cells isolated from samples at 2 dpa. Differential gene expression showed the up-regulation of injury responsive genes (*cebpb*, *cebpd*, and *socs3a*) and immune-related genes (*c9*, *cxcl14*, and *il4r.1*) in Hybrid 1 cells, while *pdx1* expression peaks in Hybrid 2 cells (fig. S15E). The expression levels of *ins* and *mnx1*, however, are most highly expressed in Beta 1 cell (fig. S15E). Regulon analyses identified 16 regulons; the Beta 1/2 cells can be easily distinguished from Hybrid 1/2/3 cells based on the regulons (fig. S15F). We also demonstrated that the estrogen receptor *esr1* regulon dominates in the two clusters of mature β cells derived from samples in the basal state (fig. S15G). On the basis of the regulon, the β cells can be very well separated from the hybrid cells, indicating that the transcriptional state of the hybrid cells are distinct from the β cells (fig. S15, H and I). We also observed that both *fev* expression and fev regulon are up-regulated in the Hybrid cells, highlighting the function of *fev* in driving cells toward a precursor-like multihormonal state (fig. S15J), which indicate similar transcriptional regulation in cellular conversion and neogenesis.

## DISCUSSION

**Fig. 9.**
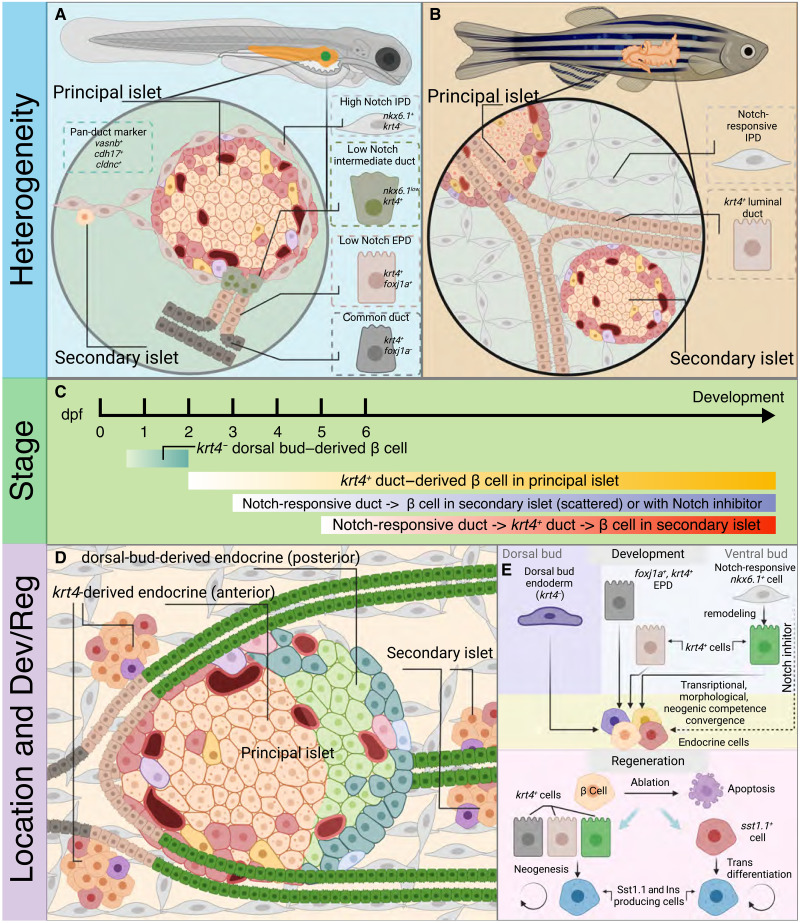
Graphical summary describes the landscape of endocrinogenesis in zebrafish pancreas. (**A**) Illustration of zebrafish pancreas in larval and juvenile/adult stage. The schematic showing that in larval stage, the zebrafish ductal tree is composed of extrapancreatic duct (EPD) (*foxj1a^+/^krt4^+^*), intermediate duct (*foxj1^−/^krt4^+^*), and Notch-responsive intrapancreatic duct (IPD) (*tp1^+/^krt4^−^*); while in juvenile and adult, the *krt4^+^* duct forms luminal structure extending to the pancreatic tail, which also contain Notch-responsive ductal cells located in the peripheral regions. (**B**) The sketch of zebrafish endocrinogenesis suggesting multiple phases of neogenesis that take place at various stages. (**C**) Schematic summary of the divergent origins and contributions of the *krt4^+^*duct in juvenile and adult pancreata. (**D** and **E**) The working model of β cell differentiation and insulin-producing cell regeneration. During development, the primitive islet is formed from the dorsal pancreatic bud, followed by *krt4-*derived duct-to-endocrine differentiation in the principal islet. The Notch-responsive duct does not naturally serve as the direct progenitor of endocrine cells but gradually undergoes transformation and assembles to become *krt4^+^* luminal duct, which has neogenic competence and can differentiate into endocrine cells in the secondary islets throughout the development until the adult stage. After massive loss of β cells, the *neurod1^+^/sst1.1^+^* endocrine cells would initiate reprogramming and up-regulate insulin expression for functional compensation.

By leveraging single-cell transcriptomics and a multitude of novel knock-in and transgenic lines for lineage tracing and targeted cell ablation, our study reveals several major discoveries and proposes a redefined model of the development and regeneration of endocrine cells in zebrafish (Fig. 9). First, we unraveled ductal heterogeneity in the zebrafish pancreas and found that the *krt4^+^* extrapancreatic and intermediate ducts (in larvae) and all luminal ducts (in a continuum throughout pancreatic tail in juveniles and adults) serve as the direct source of endocrine cells. This finding is in agreement with the previous lineage tracing study shown by spatiotemporal labeling using *Tg(ins:CreERT2, cryaa:Venus)^KI138^* [abbreviated as *Tg(ins:CreERT2)*] and confirmed the hypothesis of the existence of unknown progenitor cells residing anterior to the principal islet (*30*). In this regard, *anxa4* has been proposed to mark plastic duct anterior to the principal islet in larvae, but since *anxa4* is also expressed in endocrine cells, it is unusable for lineage tracing experiments (*58*, *59*) despite having a similar ductal expression to *krt4* (at least in embryonic stages). Further studies are needed to clarify differences between *anxa4*^+^ and *krt4*^+^ ductal compartments in various stages of development and the adult. Nevertheless, our current study provides extensive and novel molecular and cellular insights that add to the mechanistic understanding of the *krt4*^+^ duct and its plasticity. It also expands the prior discoveries showing the dynamics of extrapancreatic duct–to–β cell differentiation based on time-lapse imaging and BMP/fibroblast growth factor signaling inhibition described in the embryonic stage (*49*, *58*, *60*). Moreover, we revealed a ductal cell remodeling process occurring in developed juvenile fish, indicated by complementary temporally controlled fate mapping experiments.

The divergent sources of ductal cells found at different stages can be gradually reprogrammed to transcriptionally and morphologically similar states and retain the capability to differentiate. Such a model has previously been implicated in oligodendrocyte precursor cells in mouse development, indicating a universal principle across different organs and species (*61*). In addition, these experiments resolved fundamental questions regarding duct-to-endocrine neogenesis in zebrafish. The prevailing perspective suggests that the *tp1*-driven Notch-responsive ductal cells, also known as centroacinar cells, are the progenitors of endocrine cells (*32*, *33*, *42*, *43*). However, the *tp1*-driven lineage tracing results either labeled a minority of the regenerated β cells or was without temporal control and not specific to the Notch-responsive ductal cells (expression of which could be induced either in the intrapancreatic duct or the differentiated cells) (*30*, *32*, *43*). In our revised model, we reconcile the old model with the new knowledge presented in the current study. We propose that Notch-responsive intrapancreatic ductal cells (with a fibroblast-appearing morphology) can be naturally reprogrammed to form *krt4^+^* luminal ducts throughout the pancreas, and, thus, a subset of endocrine cells in the secondary islets can be traced back to the Notch-responsive duct and a *nkx6.1^+^* cell origin (*32*, *62*). These observations are in concordance with ductal remodeling in the liver upon lumen formation and Notch-signaling deficiency (*63*–*65*). Further, there are other *krt4^+^* ductal cells from surrogate sources, i.e. extrapancreatic and intermediate duct, that account for the endocrine cell formation in the anterior part of principal islet. We observed that the Notch-responsive ducts and *krt4^+^* luminal ducts have marked differences in transcriptional states, in terms of the Notch-target genes, with *her15.1/her15.2* being specifically expressed in the Notch-responsive duct and *her6/her9* mainly in the *krt4^+^* duct. These differential Notch signatures reflect a complex Notch activation pattern and might explain the limited effects of Notch inhibition on *krt4^+^* duct cells using canonical γ-secretase inhibitors. Further investigation is needed to unveil the distinct roles of these Notch-target genes in ductal cell identity maintenance and progenitor cell property settlement.

Using several different Cre/CreERT2 lines, we provide concrete evidence showing multiple coordinated cellular behaviors during β cell regeneration. Our comprehensive analyses address multiple potential cell sources of regenerative insulin–producing cells and provide detailed lineage paths of endocrine cell transdifferentiation and duct neogenesis of β cells during regeneration. We applied a temporally controlled lineage tracing strategy (based on *neurod1*-driven expression of CreERT2) to consolidate the concept of endocrine cell transdifferentiation serving as the major source of regenerative insulin–producing cells (*37*, *39*). Combining various Cre lines to track specific endocrine cell types, we ruled out α-to-β, δ-to-β, and gip-to-β conversion as the predominant contributors. We also showed that both *neurod1*- and *krt4*-derived regenerative insulin–producing cells coexpress Sst1.1 (the ortholog of mouse and human Sst), indicating that a distinct gene regulatory network was reconstructed in the unique regenerative microenvironment. The comparative analysis of gene regulatory networks between bona fide β cells and regenerative β cells indicated that the esr1 (estrogen receptor 1) regulon might play a pivotal role in functional maturation of β cells. This is markedly interesting as recent integrative single-cell RNA-seq data also showed a specific expression pattern of ESR1 in only adult human β cells, indicating a potential cross-species conservation in β cell maturation (https://singlecell.broadinstitute.org/single_cell/study/SCP1526/functional-metabolic-and-transcriptional-maturation-of-human-pancreatic-islets-derived-from-stem-cells) (*66*). Moreover, Esr1 has been intensively investigated in rodent models and is shown to play essential roles in β cell iron metabolism, promoting β cell survival, preventing β cell apoptosis, and maintaining mitochondrial and endoplasmic reticulum functions (*67*–*73*). Functional assays, novel in vivo models or 3D cocultured systems are needed to reveal the environmental cues that drive the differential transcriptional states in between the mono- and bihormonal cells and figure out the permissive niche signals to promote β cell maturation.

To dissect the lineage path at the molecular level, we performed single-cell RNA-seq using pancreata isolated from adult zebrafish in the physiological state and in a series of time points during regeneration. We comprehensively characterized the molecular signatures of the *krt4* lineage cells using several algorithms and managed to delineate a conserved lineage path, i.e., the transition-to-*ins/sst1.1* hybrid cells. Notably, we found that the *dlb^+^* cells demonstrated a unique transcriptional state featured by up-regulation of multiple lineage-committed transcription factors (*myt1a*, *fev*, and *pax4*), as well as a multihormonal expression pattern (*ins*, *sst1.1*, *gcga*, *gcgb*, *sst2*, and *ghrl*). In addition, these cells specifically express the Notch ligand *dlb* and up-regulate the Notch negative regulator *hes6*, indicating that Notch inhibition and lateral inhibition might be important events involved in *krt4^+^* duct-to-endocrine conversion. We note that *dlb* is the zebrafish ortholog of the human DLL1 gene. These results are consistent with previous findings in mouse and human embryonic pancreas development, with genes featured in trunk EPs, including *Hes6* and *Dll1*, getting up-regulated in the EP stage, followed by downregulation during further endocrine differentiation (*6*, *7*). Although it is still under debate whether adult mice harbor pancreatic ductal progenitors, our results nevertheless suggest an evolutionarily conserved duct-to-endocrine differentiation between adult zebrafish and embryonic mouse and human (*22*, *25*, *74*, *75*).

The *dlb^+^* cells also maintain a certain level of epithelial cell markers, such as *cdh17*, *cldnh*, *cldnc*, and *epcam*, suggesting that they are transitional between *krt4^+^* duct and endocrine cells. These transcriptional features implicate these cells as being in a primed state for further differentiation to endocrine cells. Our regulon analyses showed that these *dlb*^+^ cells are enriched with atf3, fev, crema, and creb3l1 regulons. *Neurog3* and *Fev* have been recognized to play sequential roles in mouse endocrinogenesis (*3*, *7*), and our study suggests that the endocrine progenitor cell state in zebrafish is also governed by *fev*. The *sst1.1^+^* endocrine cell conversion to insulin-producing cells also involves the up-regulation of *fev* (regulon) to form hybrid cells. Thus, these results highlight the conserved functional role of *fev* in endocrinogenesis and its distinct function in direct reprogramming (*76*, *77*). Whether the hybrid cells resolve into monohormonal cells could be dependent on their origin and timing; when we ablated the β cells in larvae, we observed mostly monohormonal *krt4* lineage–traced cells in the juvenile stage, whereas when Carril Pardo *et al.* (*39*) ablated β cells in the adult, the *sst1.1*-derived hybrid cells remained bihormonal. Thus, it appears that the origin of insulin-producing cells, i.e., from *krt4^+^* duct in development/growth versus *sst1.1^+^* cells after β cell ablation, has different abilities to become monohormonal.

Our velocity-based analyses further revealed that both dedifferentiation and differentiation processes occur during recovery from β cell loss, although dedifferentiation seems to dominate this dynamic process. We identified two late transitional cell states that, in the trajectory, sit in between the *dlb^+^* cells and the regenerative insulin–producing cells. The cells showing up-regulation of various immune responsive genes are only enriched in the acute phase after two consecutive rounds of β cell ablation. A subset of these cells had more unspliced than spliced insulin mRNA, with velocity vectors pointing toward the insulin-producing cell fate. These cells also up-regulated EMT-related genes, indicating an ongoing differentiation process (*78*). The cells showing dedifferentiation vectors up-regulate a broad collection of epigenetic remodeling genes (*79*). These results suggest that the inflammatory response following β cell loss might play an essential role in the course of differentiation. We speculate that the dysregulation of epigenetic factors might serve as either a protective factor in response to cellular aging/stress or a detrimental factor in preventing insulin-producing cell maturation. Considering the latter case, in combination with our regulon results, reversing the behavior of these cells by targeting the key regulons might be a promising strategy to promote redifferentiation and increase functional β cell mass (*79*). We noted that a wide variety of activating protein 1 (AP-1) genes (*jun*, *jund*, *junba*, and *fosb*) are highly expressed in the early transition cells. Since the AP-1 complex has been shown to function in the signal transduction of proregenerative responses in numerous tissues (e.g., zebrafish and salamander ependymo-radial glia, mouse subventricular zone, and zebrafish lateral line hair cells), we propose that the inflammatory response could provide a favorable microenvironment to trigger progenitor cells to differentiate (*56*, *80*–*86*). This is supported by the regulon analysis showing that the injury responsive genes (*cebpa* and *cebpd*) and regulons are dominating in late transition 1 cells. However, further functional studies are needed to unveil whether and how much inflammatory signaling is required to promote induction of endocrine cells upon injury (*87*).

We also applied pharmaceutical approaches to investigating the potential pathways that are crucial for differentiation to insulin-producing cells. By repressing the canonical PI3K-Akt-mTOR pathway using wortmannin, we observed an increase in *krt4*^+^ lineage–traced β cells in the basal state, suggesting that PI3K signaling regulates cell differentiation or maturation (*57*).

Last, we would have been unable to make these discoveries without developing novel knock-in zebrafish tools. The previously published findings in zebrafish were based on classical transgenic lines that could be compromised by their natural disadvantages, such as the ectopic labeling (transgene leakage issue) or a lack of labeling. In particular, we need to be cautious in drawing definitive conclusions when only using reporters of signaling pathways. This is due to the complexity of signaling pathways (a wide range of downstream responsive genes responds differently to various environmental stimuli) making it questionable whether the cloned sequence elements can recapitulate the complex activation pattern of a signaling pathway. Our study serves as proof of concept for using a new generation of knock-in tracers (targeting key transcription factors or structural proteins) to resolve lineage-related questions in zebrafish. This could lead to a new standard of genetic fate mapping for both confirming old and generating new discoveries in the zebrafish field.

In conclusion, our study provides solid evidence for a new model of β cell differentiation and recovery in zebrafish. We identify a ductal remodeling process that fills the gaps in the previous model. The comprehensive molecular profiling at the single-cell level suggested both distinct and conserved aspects of β cell neogenesis in zebrafish, highlighting the advantage of using zebrafish and opening new avenues for understanding and treating human diseases.

## MATERIALS AND METHODS

### Zebrafish lines and husbandry

The following published transgenic and knock-in zebrafish lines were used in this study: *Tg(Tp1bglob:H2BmCherry)*^S939^ (*43*) abbreviated as *Tg(tp1:H2BmCherry)*, *Tg(tp1:EGFP)* (*43*), *Tg(tp1:CreERT2)* (*41*), *TgKI(krt4-p2A-mNeonGreen-t2A-iCre)^KI128^* (*47*) abbreviated as *TgKI(krt4:iCre)*, *TgKI(krt4-p2A-EGFP-t2A-CreERT2)^KI129^* (*47*) abbreviated as *TgKI(krt4:CreERT2)*, *TgKI(krt4-p2a-mNeonGreen)^KI127^* (*47*), *TgKI(nkx6.1:CreERT2)* (*47*), *TgBAC(cftr:Gal4)^pd1101^* (*88*), *Tg(UAS:GFP)^zf82^* (*89*), *Tg(5xUAS:EGFP)^nkuasgfp1a^* (*89*) abbreviated as *Tg(UAS:Cre)*, *Tg(-3.5ubb:loxP-EGFP-loxP-mCherry)^cz1701^* (*90*) abbreviated as *Tg(ubi:Switch)*, *Tg(UBB:loxP-CFP-STOP-Terminator-loxP-hmgb1-mCherry)^jh63^* (*64*) abbreviated as *Tg(ubi:CSHm)*, *Tg(ins:flagNTR)* (*29*), *TgBAC(neurod1:EGFP)^nl1^* (*91*), *TgBAC(pdx1:EGFP)^bns13^* (*92*), *TgBAC(ascl1b:EGFP-2A-Cre-ERT2)^ulg006^* (*62*) abbreviated as *Tg(ascl1b:EGFP)*, *Tg(nkx2.2a:mEGFP)^vu17^* (*93*), *Tg(ins:Hsa.HIST1H2BJ-GFP)^s960^* (*94*) abbreviated as *Tg(ins:H2BGFP)*, *TgBAC(arxa:Cre)^bns250^* (*92*), *Tg(sst2:Cre)* (*34*), and *Tg(Hsa.CTGF:NLS-mCherry)^ia49^* (*95*), as referred as *yap* reporter.

Several lines were reestablished by reinjecting the following constructs: *Tg(cdh17:H2Bmcherry)* (plasmid from B. He) (*96*), and *Tg(−5.2foxj1a:EGFP)* abbreviated as *Tg(foxj1a:EGFP)* (plasmid from B. Ciruna) (*97*).

The strategy of generating *TgKI(onecut1-p2a-EGFP-t2a-CreERT2)^KI145^* and *TgKI(hes6-p2a-EGFP-t2a-CreERT2)^KI146^* lines has been described elsewhere, using polymerase chain reaction (PCR)–amplified 5′-AmC6–modified double-stranded DNA as the donor (*47*). The single-guide RNA (sgRNA) and primers used for generating the knock-in were the following: sgRNA, GCACTTGTACCAAAGCATGA(TGG) (onecut1) and AGGTGAGCATCTGTCTACCA(GGG) (hes6); *onecut1*, GCCAACTCCTCCTCCTCCAGCACTTGTACCAAAGCAGGAAGCGGAGCTACTAACTTCAGC (forward) and TTTTTTCTTTTTTTCCCCACAATGCAGCCGTTACTCCATCAAGCTGTGGCAGGGAAACCC (reverse); *hes6*, ACATTATACACCAACAAGTCCATTTGGAGACCCTGGGGAAGCGGAGCTACTAACTTCAGC (forward) and ACTTGAATGTTCATGAATCGGCGGAGGTGAGCATCTGTCTAAGCTGTGGCAGGGAAACCC (reverse).

The following lines were newly generated by the Tol2 transposon system *Tg(neurod1:CreERT2)*, *Tg(ins:CreERT2)*, and *Tg(foxj1a:iCre; cryaa:Venus)^KI139^* abbreviated as *Tg(foxj1a:iCre).* The injected constructs were built by Multisite Gateway cloning using LR Clonase II Plus (Thermo Fisher Scientific). We followed the protocol as described by C. Mosimann (dx.doi.org/10.17504/protocols.io.b4xdqxi6) of recombining entry clones p5E, pME, and p3E into a destination vector, pDEST. The following entry clones were used in this study: p5E: *p5E-ins*; *p5E-neurod1* (gift from A. Nechiporuk), *p5E-foxj1a* (gift from B. Ciruna), pME: *pME-iCre*, *pME-CreERT2*, p3E: *p3E-poly A*, and pDest: *pDEST-Tol2-gy*. The mosaic F0s and founders were selected on the basis of yellow eye marker at 3 dpf.

The following lines were newly generated by the I-*Sce*I system: *Tg(ins:loxP-dsRed-terminator-stop-loxP-DTA)^KI140^* abbreviated as *Tg(ins:CSDTA)*, *Tg(gip:iCre)^KI136^*, and *Tg(ins:CSHm)*. The double-stranded DNA sequence of DTA was synthesized by Integrated DNA Technologies. The final constructs were generated by In-Fusion cloning (Takara Bio). The mosaic F0s and founders were selected on the basis of the fluorescent signals in the islets at 3 dpf. *Tg(gip:iCre)^KI136^* was generated by replacing *Venus* with *iCre* in *Tg(gip:Venus)* using In-Fusion cloning (*98*).

Males and females aged between 3 months and 2 years were used for breeding. Zebrafish larvae were incubated at 28.5°C in E3 medium (5 mM NaCl, 0.17 mM KCl, 0.33 mM CaCl_2_, and 0.33 mM MgSO_4_ at pH 7.4 with NaHCO₃) until 6 dpf. Juvenile and adult fish were maintained on a 14:10-hour light/dark cycle at 28°C. All studies involving zebrafish were approved by the Karolinska Institutet Animal Care and Use Committee, performed in accordance with local guidelines and regulations, and approved by regional authorities.

### Lineage tracing with or without tamoxifen-inducible Cre recombinase

We used both Cre and CreERT2 lines for genetic fate mapping. The knock-in Cre lines were crossed with *Tg(ubi:CSHm)* for the quantitative analyses. For a more strict approach with temporal labeling, we treated the zebrafish larvae carrying the CreERT2 cassette as well as the *ubi:CSHm* or *ubi:Switch* transgene with 20 μM 4-OHT unless otherwise stated (Sigma-Aldrich) in E3 medium in 24-well plates, with four to eight embryos/larvae per well, for 24 hours without refreshment to accomplish maximum labeling without compromising the survival rate. We treated *TgKI(nkx6.1:CreERT2)* and *TgKI(krt4:CreERT2)* with 4-OHT from 1 to 2 dpf, while we performed the treatment for *Tg(tp1:CreERT2)*, *Tg(ins:CreERT2)*, and *Tg(neurod1:CreERT2)* from 2 to 3 dpf.

### Sample fixation for immunostaining

Before fixation, we euthanized the zebrafish with tricaine (Sigma-Aldrich) in E3 medium, followed by washing three times with distilled water. We then fixed the samples in 4% formaldehyde (Sigma-Aldrich) in phosphate-buffered saline (PBS) (Thermo Fisher Scientific) at 4°C for at least 24 hours. We stopped the fixation by washing three times with PBS and then removed the skin, crystallized yolk (of larvae), and liver (if needed) to expose the pancreas for immunostaining.

### Immunostaining

We followed the immunostaining protocol that was previously described by Liu *et al*. (*99*). Briefly, we incubated the samples in blocking solution [0.3% Triton X-100 and 4% bovine serum albumin (BSA) in PBS] at room temperature for at least 1 hour, followed by overnight incubation with primary antibodies at 4°C. After removing the primary antibodies, we washed the samples using washing buffer (0.3% Triton X-100 in PBS) at least 10 times at room temperature. Next, we incubated the samples in blocking solution with fluorescent dye–conjugated secondary antibodies and the nuclear counterstain 4′,6-diamidino-2-phenylindole (DAPI) (Thermo Fisher Scientific) at 4°C overnight. On the following day, we removed the solution and washed the samples with washing buffer 10 times at room temperature. The following primary antibodies were used: chicken anti-GFP (1:500; Aves Labs, GFP-1020), goat anti-tdTomato (1:500; MyBioSource, MBS448092), rabbit anti-insulin (1:100; Cambridge Research Biochemicals, customized), mouse anti-glucagon (1:50; Sigma-Aldrich, G2654), rat anti-Sst1.1 (1:50; GeneTex, GTX39061), rabbit anti-Cdh17 (1:1000; customized sera, gift from Y. Cao, Tongji University), rabbit anti-Cldnc (1:200; customized sera, gift from Y. Cao, Tongji University), rabbit anti-Vasnb (1:1000; customized sera, gift from P. Panza), and rabbit anti-activated Caspase-3 (1:100; Chemicon).

### Hybridization chain reaction

HCR3.0 was performed following the protocol described by Choi *et al*. (*100*). Briefly, the larvae were fixed in 4% paraformaldehyde (PFA) at 4°C overnight, with subsequent perforation step in 100% methanol at −20°C for at least 24 hours. After that, we performed gradient rehydration steps using 75, 50, and 25% methanol, followed by washing five times using 100% PBS with 0.1% Tween 20 (PBST). The larvae were then treated with a series of preparation steps including further permeabilization [using proteinase K (30 μg/ml) for 45 min at room temperature], postfixation (4% PFA for 20 min), five washes with PBST, and prehybridization (using hybridization buffer at 37 °C for 30 min). The larvae were then incubated overnight at 37 °C in the hybridization buffer containing 2 pmol of the probe set. On the next day, the solution was washed off four times with washing buffer at 37°C, followed by 4× saline‑sodium citrate with 0.1% Tween 20 (SSCT) washes at room temperature for 15 min each. Next, the larvae were incubated in the amplification buffer with 15 pmol of fluorescently labeled hairpin amplifier overnight at room temperature. On the next day, the larvae were washed twice with 5× SSCT, followed by one incubation step with DAPI (1:1000; in 5× SSCT) for 30 min at room temperature. Last, we performed additional washes three times with 5× SSCT. Probe sequences were designed by the manufacturer (Molecular Instruments). The probe sets used were as follows: dr_krt4-B1, dr_cftr-B1, dr_sox9b-B1, dr_hnf1ba-B1, dr_cx30.3-B1, dr_clcn1b-B1, dr_cldn7b-B1, dr_cd9d-B1, dr_igf2a-B1, dr_anxa4-B1, dr_id2a-B1, and dr_foxj1a-B1. The conjugated hairpin amplifier was B1, with the fluorophore 647.

### Confocal imaging

Before confocal imaging, we mounted the stained samples in VECTASHIELD Antifade Mounting Medium (Vector Laboratories) on microscope slides with the pancreas facing the cover slips. We imaged the pancreas with the Leica TCS SP8 platform using 40× objectives for larvae until 7 dpf and 20× objectives for juveniles later than 30 dpf. The images were analyzed by Fiji (*101*).

### Chemical ablation of β cells and drug treatment

Ablation of β cells in the zebrafish larvae was performed by incubating the larvae carrying the *ins:flagNTR* transgene for 24 hours with 10 mM MTZ (Sigma-Aldrich) diluted in 1% dimethyl sulfoxide (VWR) in E3 solution supplemented with 0.2 mM 1-phenyl-2-thiourea (Acros Organics) from 3 to 4 dpf. For the juvenile and adult stages, β cell ablation was achieved by incubating the zebrafish with 1 mM MTZ for 24 hours. Chemical treatment of zebrafish larvae was performed by adding the chemicals to E3 buffer for 48 hours unless otherwise stated. The chemicals used were 50 nM wortmannin (Selleckchem, S2758) and 1 μM LY-411575 (Selleckchem, S2714).

### Adult zebrafish pancreas dissection and FACS

We euthanized the adult fish by putting each one into ice-cold Hanks’ balanced salt solution (HBSS; no calcium and no magnesium) for 10 min. Next, we used blunt forceps to carefully remove the skin, kidney, eggs (in females), liver, and gallbladder to expose the pancreata. This step is critical for avoiding *krt4^+^* cell contamination from other organs. We also removed adipose tissue as much as possible. Then, we cut at the anterior region of the intestinal bulb and hindgut and transferred the intestine and pancreas together to a new dish. We used blunt dissection tools to carefully separate the pancreata from the intestine. Meanwhile, we also removed the spleen (appearing in dark red), which is also attached to the intestine. We moved and immersed the whole pancreata into 5 ml of HBSS kept on ice. For each condition, we pooled four to eight samples together for an enzymatic digestion with 600 μl of 1× TrypLE (Thermo Fisher Scientific) supplemented with 60 μl of 100× Pluronic F-68 (Thermo Fisher Scientific) at 37°C on a shaker at 125 rpm for 45 to 60 min to prevent tissue adhesion. We also pipetted the tissue up and down every 5 min for better digestion. We added 6 ml of precooled 2% BSA to end the digestion and centrifuged the sample at 500*g* for 5 min. After removing the supernatant, we washed the pellets with 300 μl of precooled solution containing 1% BSA, 0.1% Pluronic F-68, and 0.1% DAPI and used a Corning Falcon cell strainer (Corning, 352235) to filter out not fully digested tissues.

During FACS, we selected single-cell populations based on forward scatter and side scatter signals. Next, we performed negative selection with the DAPI channel to remove dead cells and cell debris. Last, we used the Cherry channel for the subsequent gating to collect all cells of the *krt4-*derived lineage in a new tube as the single-cell suspension.

### Cell encapsulation, library preparation, and sequencing

Droplet-based single-cell RNA-seq was performed using the Chromium Single Cell 3′ Library and Gel Bead Kit v3 (10× Genomics) and Chromium Single Cell 3′ Chip G (10× Genomics). Approximately 8000 to 10,000 cells from each condition were loaded and encapsulated in a single v3 reaction. GEM generation and library preparation were performed according to manufacturer’s instructions. Cells were partitioned into gel beads in emulsion in the controller for cell lysis and reverse transcription. The 10× single-cell RNA-seq libraries were PCR-amplified (13 cycles), pooled, denatured, and diluted in before paired-end sequencing on a NextSeq 500 according to manufacturer’s recommendations. Sequencing data were aligned to the zebrafish reference genome (GRCz11) with addition of the mCherry sequence using Cell Ranger v5.0.1 (10× Genomics) to generate a gene-by-cell count matrix with default parameters.

### Data preprocessing

The single-cell RNA-seq data of adult zebrafish principal islet datasets were downloaded from the Gene Expression Omnibus under accession numbers GSE106121 and GSE166052. The unique molecular identifier (UMI) counts matrix was imported into R and processed using the Seurat R package version 4.0.4 (*102*). Cells with a detected gene number of <400 or above 7000 genes were considered as low-quality cells or doublets and removed before downstream analysis. The count matrix was then normalized and scaled with default parameters, followed by highly variable gene selection. Further doublet filtering was performed using the Doubletfinder R package (*103*). After regular dimension reduction using PCA, we selected 8 to 30 PCs (depending on the anticipated cell types) for graph-based clustering (Louvain). We used the UMAP algorithm to display the relationships within and between different clusters and the Harmony algorithm for batch correction (*104*). To unravel the molecular details and cellular trajectory in *krt4*-derived duct-to-endocrine and endocrine lineages, we manually annotated each cell cluster based on well-known verified marker genes and made subsets of clusters of interest, followed by a series of normalization, scaling, dimensional reduction, batch correction, and graph-based clustering. The lists of genes encoding DNA binding proteins, membrane proteins, cell junctions, and mitochondrial proteins are retrieved from human protein atlas (www.proteinatlas.org/), and we used “biomaRt” R package to perform human-to-zebrafish ortholog conversion. The marker genes and genes with differential expression were listed in tables S6 to S21.

### SCENIC analysis

The SCENIC pipeline was performed using the python package pySCENIC (*105*, *106*). We first selected the 3000 most high variable genes and used their original count as input. We took advantage of the recent study in zebrafish that provided us a list of known transcription factors and the motif annotation for the construction of adjacency matrix (indicating the gene expression correlation between the transcription factors and the putative target genes) refined gene regulatory network after pruning targets (*107*). Last, cells were scored for each regulon and obtained a regulon activity score (RAS) using AUCell (www.bioconductor.org/packages/release/bioc/html/AUCell.html) (*105*). We applied the RAS matrix to generate t-distributed stochastic neighbor embedding (t-SNE) and UMAP embedding. In downstream analyses, we followed the analytical pipeline that was previously described by Suo *et al.* (*108*). Briefly, we first calculated the regulon specific score matrix to identify the cell type/state–specific regulon. We also used hierarchical clustering built on the regulon to generate a connection specificity index matrix and define individual regulon modules, which exhibit the relationship between the regulon and regulon module. Last, we measured the regulon module activity scores (average RAS on regulons in each module) for all cells and averaged them on the basis of cell type to identify the correlation between the regulon modules and cell types. The list of transcription factors and the motif annotations can be accessed from https://db.cngb.org/stomics/zesta/.

### Pseudotime trajectory and in silico fate mapping using scVelo and CellRank

Psuedotime analysis was conducted with slingshot (version 1.8.0) (*109*) and Monocle 3 (version 1.2.9) (*110*) after batch correction. We used the default settings and defined the early transition as the root node for pseudotime calculations.

scVelo was used to calculate RNA velocity in transition-to-*ins/sst1.1* hybrid cells (*5*). We first selected cells based on barcoding and filtered gene expression matrix to only include the 3000 most highly variable genes. Then, we computed the moments for velocity estimation using *n* = 15 nearest neighbors and the top 8 PCs. We calculated the latent time using the cells from the uninjured samples as root. Last, RNA velocity was estimated on the basis of latent time scores, using the dynamical model, and we mapped the velocity vectors on the prior UMAP embedding. We leveraged the directional information from RNA velocity and computed the initial and terminal states of the lineage path using the function “cr.tl.initial_states” and “cr.tl.initial_states” in CellRank with default settings (*8*).

### Enrichment analysis

We selected differentially expressed genes among different clusters found by “FindMarkers” function with parameter (min.pct = 0.25, logfc.threshold = 0.25) and filtered by pvalue.adj < 0.05. Gene enrichment analyses were accomplished using the function “enrichGO” with pvalueCutoff = 0.01, qvalueCutoff = 0.05 (OrgDb = org.Dr.eg.db, pAdjustMethod = “BH”) from the R package clusterProfiler (V3.18.1, https://bioconductor.org/packages/release/bioc/html/clusterProfiler.html) (*111*). To compare pathway activity between different cell states along transition-to-*ins/sst1.1* hybrid cell lineage, we fetched the normalized data of the groups and calculated the pathway activity score based on the 50 hallmark gene sets listed in Molecular Signatures Database (MSigDB) v7.5.1 (www.gsea-msigdb.org/gsea/msigdb/genesets.jsp?collection=H) using the function “gsva” in the GSVA R package (www.bioconductor.org/packages/release/bioc/html/GSVA.html) (*112*). The limma package was used to identify the differentially enriched pathways between the two selected groups. To decipher and visualize the pathway activity of individual cells along the transition-to-*ins/sst1.1* hybrid cell path, we also used the hallmark gene sets in MSigDB and calculated the pathway activity score of each module using function “AddModuleScore” from Seurat.

### Statistical analysis

Similar experiments were performed at least two times independently. For immunostaining and HCR3.0 in situ hybridization experiments, the replicates were performed and the expression patterns were confirmed in at least five individual samples, i.e., where only representative images were presented in the figure. For juvenile and adult lineage tracing experiments, results were confirmed in at least two independent experiments performed on different days. No statistical method was used to predetermine sample size. No data were excluded from the analyses. The number of cells in the confocal microscopy images was all quantified manually with the aid of Fiji. The scheme of knock-in strategy was illustrated by using “IBS” software (*113*). Statistical analyses were carried out by two-tailed Mann Whitney *U* tests (comparing two groups) or by Kruskal-Wallis tests (comparing three groups) unless otherwise stated. The results were presented as the mean values ± SEM, and *P* ≤ 0.05 is considered statistically significant. The n number represents the number of zebrafish in each group for each experiment. All statistical analyses and data visualization were performed on R platform (version 4.0.2) using “ggplot2” and “ggpubr” packages.
