# Supplementary material for: Decoding pancreatic endocrine cell differentiation and β cell regeneration in zebrafish
Source: Sci Adv. 2023 Aug 18;9(33):eadf5142. doi: 10.1126/sciadv.adf5142 (PMC10438462; doi:10.1126/sciadv.adf5142)
Supplement: Supplementary file 1 — Figs. S1 to S15 Legends for movies S1 to S3 Legends for tables S1 to S35 [file sciadv.adf5142_sm.pdf]

Science Advances
AAAS

Supplementary Materials for

Decoding pancreatic endocrine cell differentiation and β cell regeneration in zebrafish

Jiarui Mi *et al.*

Corresponding author: Olov Andersson, olov.andersson@ki.se

Sci. Adv. **9**, eadf5142 (2023)
DOI: 10.1126/sciadv.adf5142

The PDF file includes:

Figs. S1 to S15
Legends for movies S1 to S3
Legends for tables S1 to S35

Other Supplementary Material for this manuscript includes the following:

Tables S1 to S35
Movies S1 to S3

Additional Supplementary Material include:

Supplementary Movie 1-3. Z-stack movies showing the neogenic endocrine cells (indicated by *neurod1:EGFP)* residing in, along and closely attaching to the duct (visualized by anti-Vasnb staining and DAPI). The movies display a zoomed-out view of the pancreatic head (S1), as well as zoomed-in views of the extra-pancreatic (S2) and luminal duct in the pancreas (S3).

Supplemental tables 1-35. Listing marker genes, cell counts and proportions.

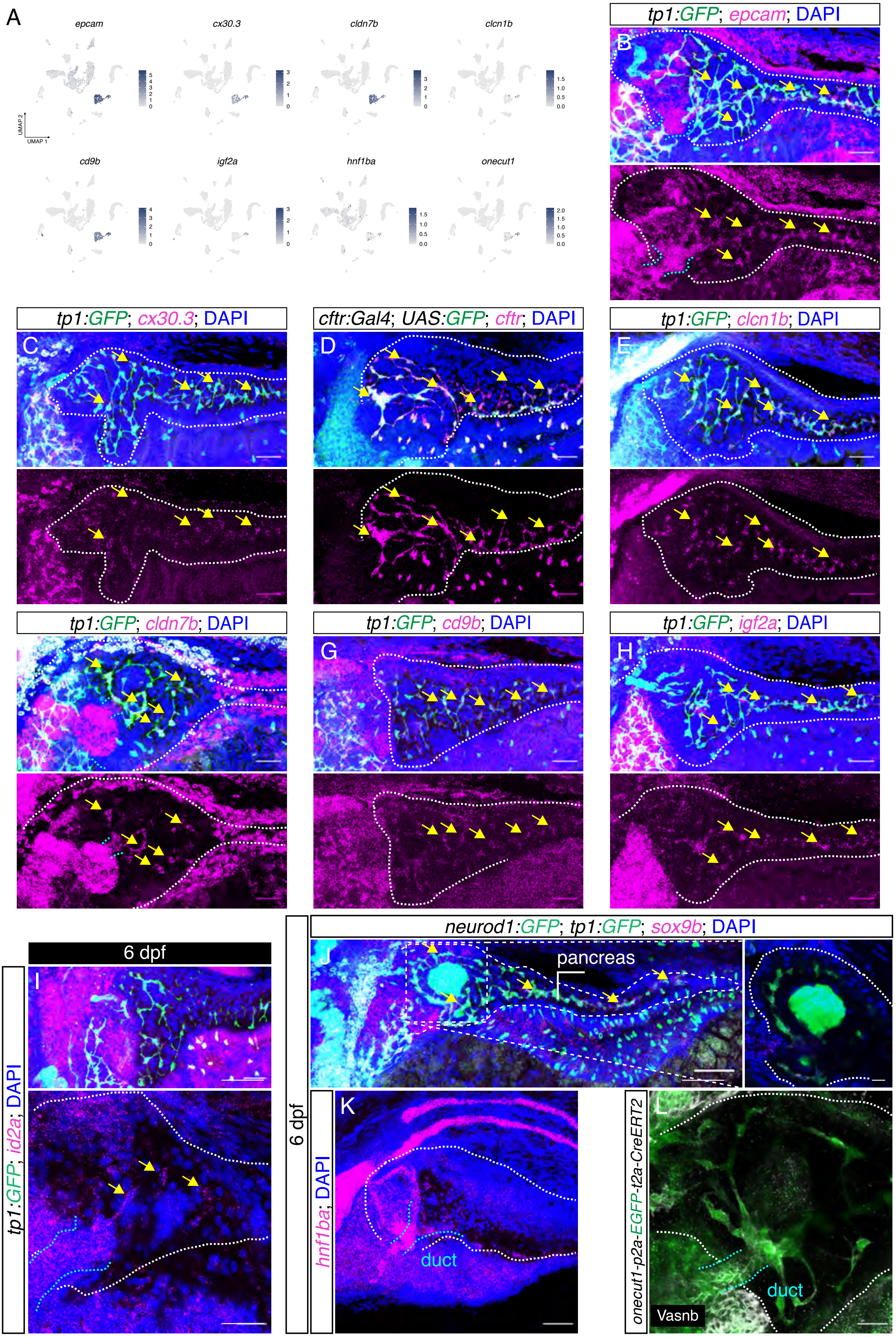

A
epcam
cx30.3
cldn7b
clcn1b
cd9b
igf2a
hnf1ba
onecut1
UMAP 1
UMAP 2
tp1:GFP; epcam; DAPI
B
tp1:GFP; cx30.3; DAPI
C
cftr:Gal4; UAS:GFP; cftr; DAPI
D
tp1:GFP; clcn1b; DAPI
E
tp1:GFP; cldn7b; DAPI
F
tp1:GFP; cd9b; DAPI
G
tp1:GFP; igf2a; DAPI
H
6 dpf
I
tp1:GFP; id2a; DAPI
6 dpf
neurod1:GFP; tp1:GFP; sox9b; DAPI
J
pancreas
K
hnf1ba; DAPI
duct
L
onecut1-p2a-EGFP-t2a-CreERT2
duct
Vasnb

Fig. S1. Characterization of ductal cells in zebrafish larvae with *in situ* hybridization.

(**A**) UMAP plot of several ductal markers indicating ductal heterogeneity. (**B**-**I**) Representative Z-projections of confocal images of HCR3.0 *in situ* hybridization (shown in magenta) for different ductal markers in *Tg(tp1:EGFP)* larvae. Scale bars = 40 μm. (**J**-**L**) Representative confocal Z-projections of HCR3.0 *in situ* hybridization of whole-mount larvae showing the expression of *sox9b* in double transgenic *Tg(tp1:EGFP);Tg(neurod1:EGFP)* larvae (**J**), as well as *hnf1ba* (**K**) at 6 dpf larvae. (**L**) Representative confocal Z-projection showing the expression of *onecut1* using *TgKI(onecut1-p2a-EGFP-t2a-CreERT2)* 6 dpf larvae. The nuclei are labelled by DAPI (blue). Scale bars = 100 μm. The white and cyan dashed lines indicate the pancreata and non-Notch-responsive duct, respectively. The yellow arrows point to intra-pancreatic ductal cells.

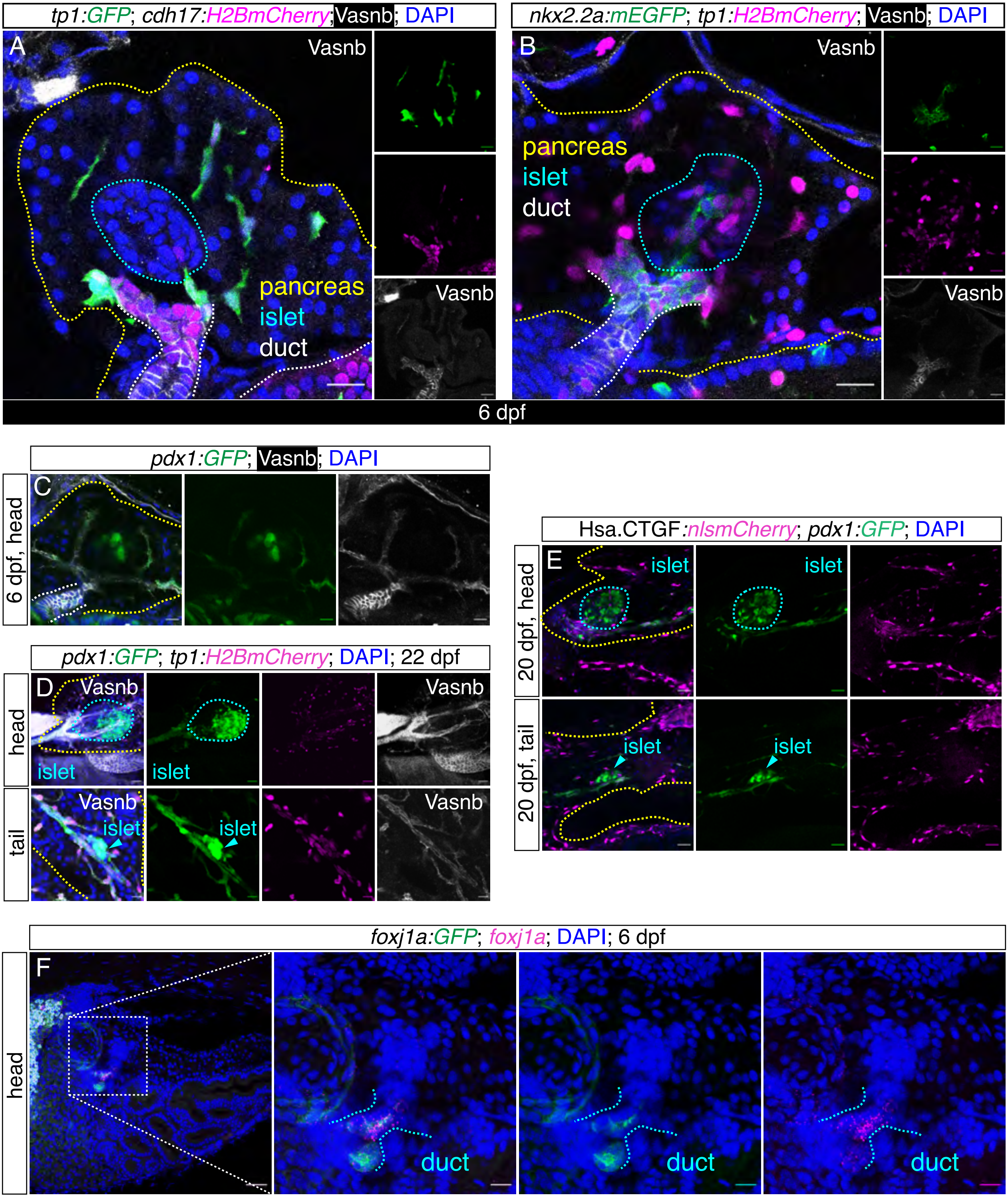
tp1:GFP; cdh17:H2BmCherry; Vasnb; DAPI
A
Vasnb
pancreas
islet
duct
Vasnb
nkx2.2a:mEGFP; tp1:H2BmCherry; Vasnb; DAPI
B
Vasnb
pancreas
islet
duct
Vasnb
6 dpf
pdx1:GFP; Vasnb; DAPI
6 dpf, head
C
Hsa.CTGF:nlsmCherry; pdx1:GFP; DAPI
20 dpf, head
E
islet
islet
pdx1:GFP; tp1:H2BmCherry; DAPI; 22 dpf
head
D
Vasnb
islet
islet
Vasnb
tail
Vasnb
islet
islet
Vasnb
20 dpf, tail
islet
islet
foxj1a:GFP; foxj1a; DAPI; 6 dpf
head
F
duct
duct
duct

Fig. S2. Characterization of ductal cells using existing transgenics. (**A**) Larvae double transgenic for *tp1:EGFP* and *cdh17:H2BmCherry* at 6 dpf. Notch-responsive cells are in green, extra-pancreatic and intermediate duct are in magenta. Anti-Vasnb staining showing the ductal tree. The yellow, cyan and white dashed lines indicate the pancreas, principal islet and the non-Notch-responsive duct, respectively. Scale bars = 40 µm. (**B**) Larvae double transgenic for *nkx2.2a:mEGFP* and *tp1:H2BmCherry* at 6 dpf. Notch-responsive cells and a subset of endocrine cells are in magenta; while a subset of intermediate ductal cells present green membrane fluorescence. Cell nuclei are in blue with DAPI staining. Anti-Vasnb staining showing the ductal tree. The yellow, cyan and white dashed lines indicate the pancreas, principal islet and the non-Notch-responsive duct, respectively. Scale bars = 40 µm. (**C** and **D**) Single-plane confocal images of *pdx1*$^{+}$ cells in *TgBAC(pdx1:EGFP)* at 6 dpf larva (**C**) and 22 dpf juvenile fish (**D**) showing the labeling pattern in intermediate and Notch-responsive ductal cells. The yellow and white dashed lines indicate the pancreas and the non-Notch responsive duct, respectively. Scale bars = 40 µm (**C**) and 100 µm (**D**). (**E**) Representative confocal Z-projection of *yap* responsive cells in the transgenic *Tg(Has.CTGF:nlsmCherry);TgBAC(pdx1:EGFP)* double transgenic juvenile fish. Scale bars = 100 µm. The yellow and cyan dashed lines indicate the pancreas and the principal islet, respectively. The cyan arrow points to the secondary islet. (**F**) *In situ* hybridization for *foxj1a* in *Tg(foxj1a:EGFP)* larva (magnified area around the principal islet are shown on the right). The cyan dashed lines indicate the non-Notch-responsive duct. Scale bars = 40 µm.

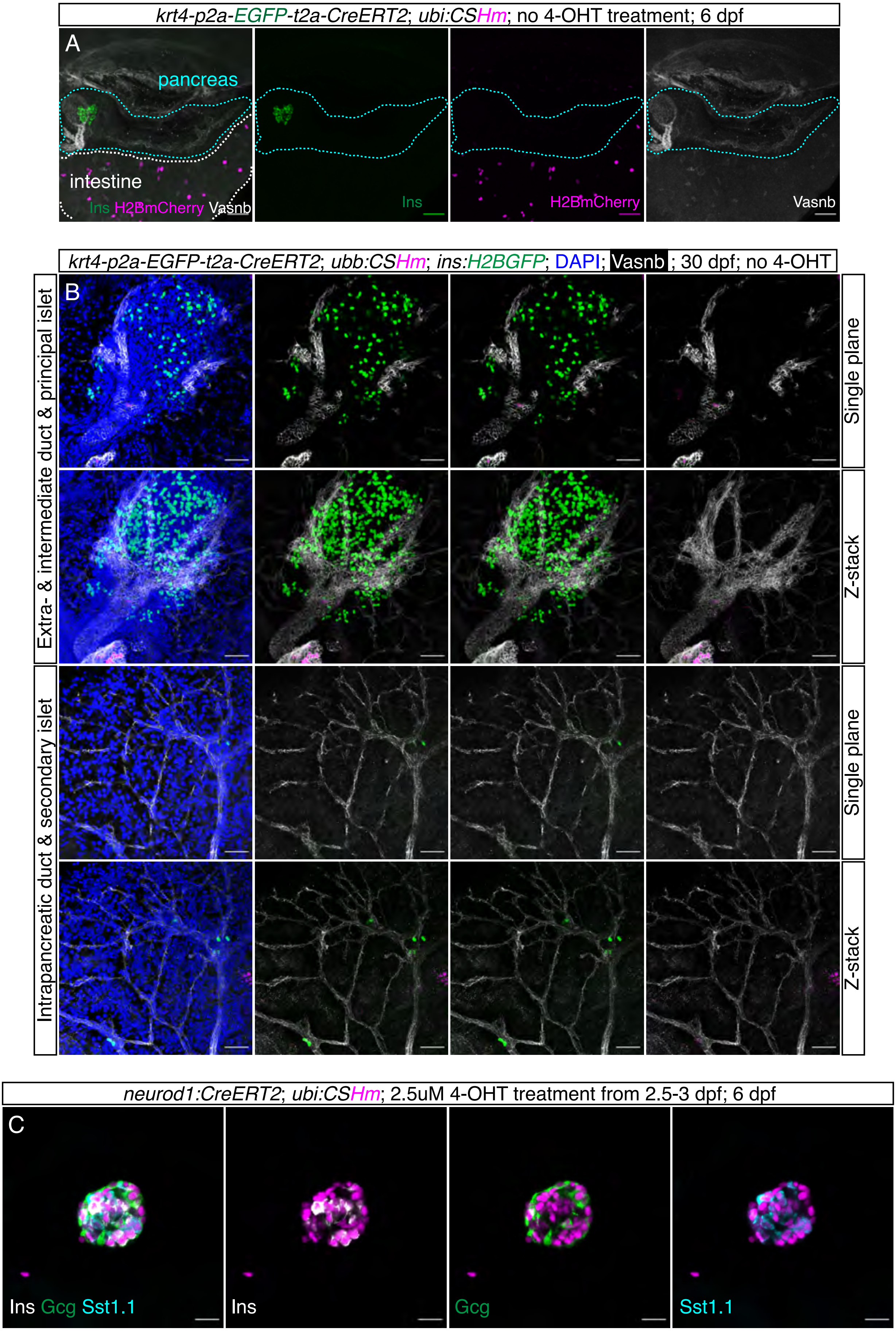

krt4-p2a-EGFP-t2a-CreERT2; ubi:CSHm; no 4-OHT treatment; 6 dpf
A
pancreas
intestine
Ins H2BmCherry Vasnb
Ins
H2BmCherry
Vasnb
krt4-p2a-EGFP-t2a-CreERT2; ubb:CSHm; ins:H2BGFP; DAPI; Vasnb; 30 dpf; no 4-OHT
B
Extra- & intermediate duct & principal islet
Intrapancreatic duct & secondary islet
Single plane
Z-stack
Single plane
Z-stack
neurod1:CreERT2; ubi:CSHm; 2.5uM 4-OHT treatment from 2.5-3 dpf; 6 dpf
C
Ins Gcg Sst1.1
Ins
Gcg
Sst1.1

Fig. S3. Control experiments confirming no ectopic leakage in the pancreatic duct using *TgKI(krt4:CreERT2)* or *Tg(neurod1:CreERT2).* (**A**) The maximum Z-projection of confocal images showing the pancreas from *TgKI(krt4:CreERT2);Tg(ubi:CSHm)* without 4-OHT treatment. The samples were stained with antibodies against Insulin (green) and Vasnb (white), as well as DAPI (blue). The cyan and white dashed lines indicate the pancreas and the intestine, respectively. Note that there is no leakage in the pancreas, but some leakage in the intestine. Scale bars = 100 µm. (**B**) Representative Z-projections of confocal images showing the pancreas in *TgKI(krt4:CreERT2);Tg(ubi:CSHm);Tg(ins:H2BGFP)* juvenile fish without 4-OHT treatment (**C**) Fish expressing *neurod1:CreERT2* are crossed to a CFP-to-H2BmCherry reporter fish under the control of ubiquitin B promoter. The H2BmCherry expression is specifically induced in *neurod1*$^{+}$ endocrine cells without ectopic leakage in ductal cells by 2.5 µM 4-OHT treatment from 2.5 to 5 dpf. Single-plane confocal image showing the labelled *neurod1*$^{+}$ cells in the pancreatic head region. The analysis was made in 6 dpf larva. Scale bars = 40 µm.

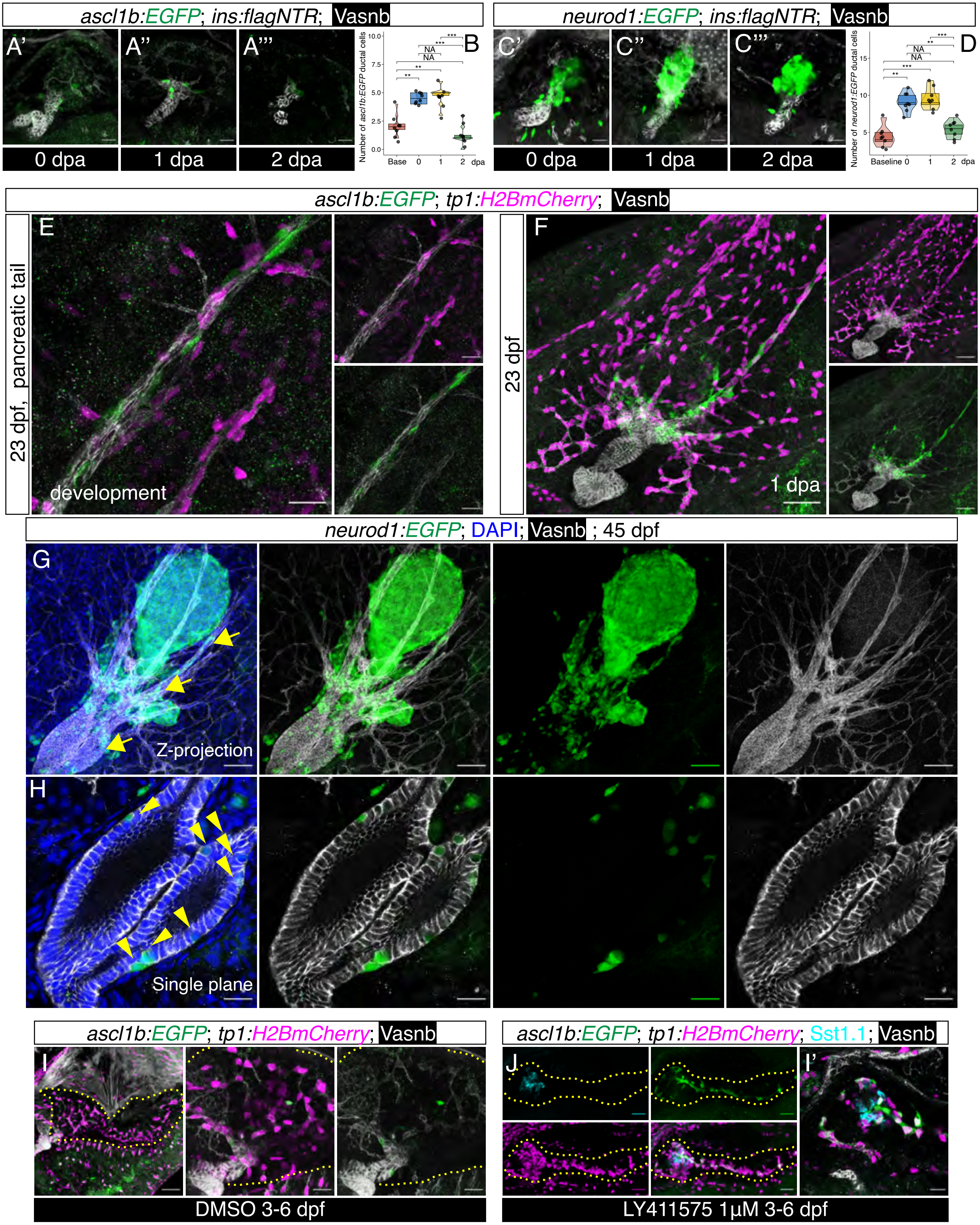
ascl1b:EGFP; ins:flagNTR; Vasnb
A'
A''
A'''
0 dpa
1 dpa
2 dpa
B
Number of ascl1b:EGFP ductal cells
Base
0
1
2
dpa
NA
neurod1:EGFP; ins:flagNTR; Vasnb
C'
C''
C'''
D
Number of neurod1:EGFP ductal cells
Baseline
ascl1b:EGFP; tp1:H2BmCherry; Vasnb
23 dpf, pancreatic tail
E
development
F
23 dpf
1 dpa
neurod1:EGFP; DAPI; Vasnb ; 45 dpf
G
Z-projection
H
Single plane
ascl1b:EGFP; tp1:H2BmCherry; Vasnb
I
DMSO 3-6 dpf
ascl1b:EGFP; tp1:H2BmCherry; Sst1.1; Vasnb
J
I'
LY411575 1μM 3-6 dpf

Fig. S4. Upregulation of endocrine progenitor transcription factors, *ascl1b* and *neurod1*, in the ductal cells in juvenile and beta-cell ablation condition. (**A**) Representative Z-projections of confocal images and quantification showing the expression pattern of *ascl1b* in/along the intermediate duct without ablation, at baseline, 0, 1 and 2 dpa. Scale bars = 40 µm. (**B**) Quantifications of EGFP positive cells per pancreas at different time points are shown in box-violin plots. Kruskal-Wallis test indicates the significant differences among the four groups with the p-value = 2.7e-5. Wilcoxon-test was used to test each pair of groups. (**C**) Representative Z - projections of confocal images and quantifications showing the expression pattern of *neurod1* in/along the intermediate duct without ablation, at baseline, 0, 1 and 2 dpa in *TgBAC(neurod1:EGFP)* larvae. Scale bars = 40 µm. (**D**) Quantifications of EGFP positive cells per pancreas at different time points are shown in box-violin plots. Kruskal-Wallis test indicates the significant differences among the four groups with the p-value = 2.5e-5. Wilcoxon-test was used to test each pair of groups. (**E** and **F**) Representative confocal Z-projections showing the expression pattern of *ascl1b* in either development (**E**) or regenerative conditions (**F)** in tail region of 23 dpf juveniles. Scale bars = 40 µm. (**G** and **H**) Z-projection and the corresponding single-plane confocal images of principal islet in 45 dpf fish. The anti-Vasnb staining was used to visualize the ductal tree. The arrows (**G**) and arrowheads (**H**) point to ductal cells overlapped with *neurod1:EGFP* signal. Scale bars = 100 µm (**G**) and 40 µm (**H**). (**I**) Control experiment showing the *ascl1b:EGFP*-expression pattern in a Z-projection of a 6 dpf larvae with DMSO treatment. (**J**) Confocal image showing the *ascl1b:EGFP*-expression pattern in Z-projection of 6 dpf larvae upon chemical-induced Notch inhibition with a single-plane magnification displayed in (**J'**). The *Tg(tp1:H2BmCherry)* together with anti-Somatostatin1.1 and anti-Vasnb stainings were used to locate and outline the co-expression pattern in the principal islet and ductal tree. The yellow dashed lines indicate the pancreas. Scale bars = 40 µm. * = p-value < 0.05, ** = p-value < 0.01, *** = p-value < 0.001.

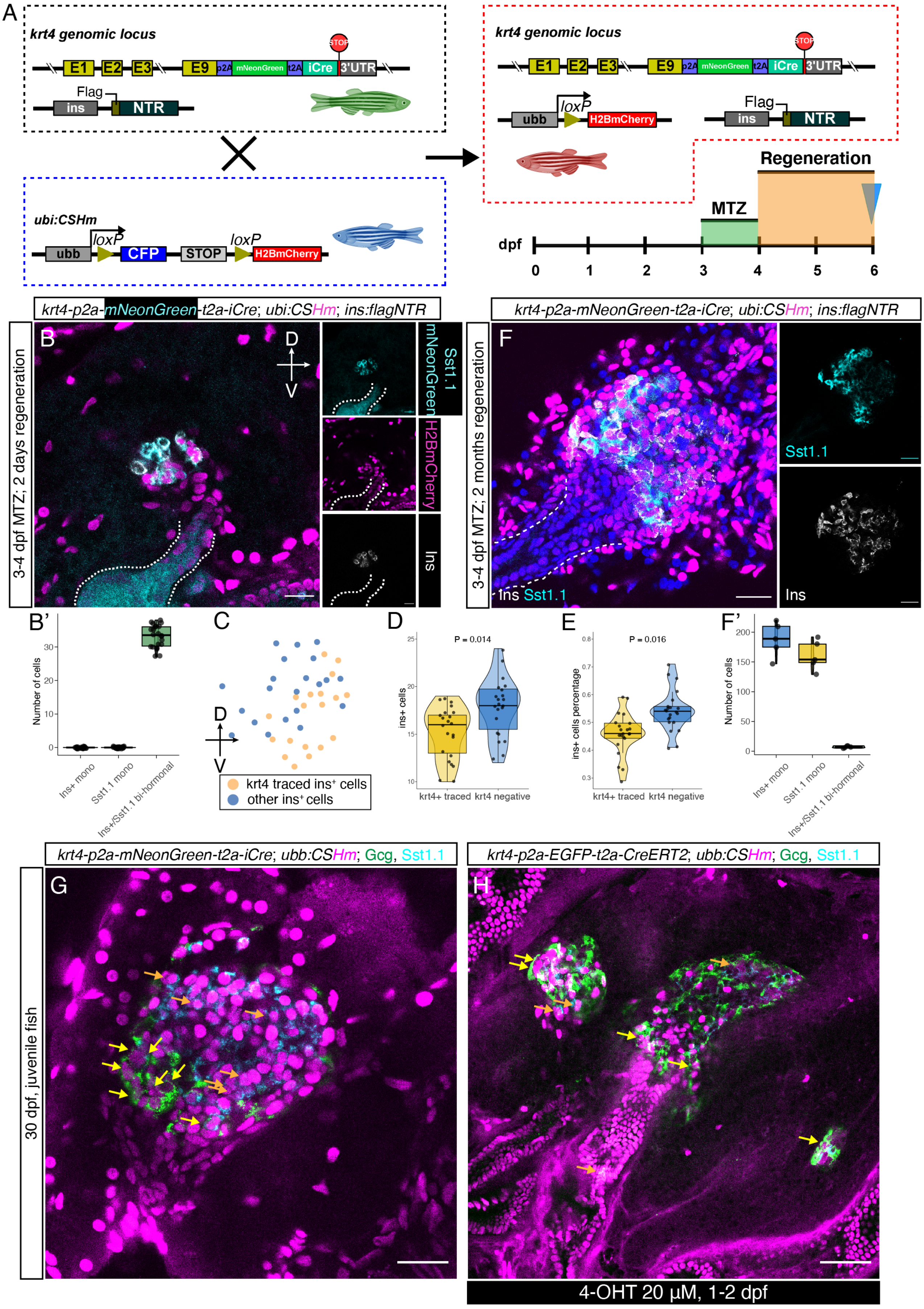
A
krt4 genomic locus
E1 E2 E3 E9 p2A mNeonGreen t2A iCre 3'UTR
STOP
Flag
ins NTR
ubi:CSHm
ubb loxP CFP STOP loxP H2BmCherry
krt4 genomic locus
E1 E2 E3 E9 p2A mNeonGreen t2A iCre 3'UTR
ubb loxP H2BmCherry
Flag
ins NTR
Regeneration
MTZ
dpf 0 1 2 3 4 5 6
krt4-p2a-mNeonGreen-t2a-iCre; ubi:CSHm; ins:flagNTR
3-4 dpf MTZ; 2 days regeneration
B
D V
Sst1.1
mNeonGreen
H2BmCherry
Ins
krt4-p2a-mNeonGreen-t2a-iCre; ubi:CSHm; ins:flagNTR
3-4 dpf MTZ; 2 months regeneration
F
Ins Sst1.1
Sst1.1
Ins
B'
Number of cells
Ins+ mono
Sst1.1 mono
Ins+/Sst1.1 bi-hormonal
C
D V
krt4 traced ins⁺ cells
other ins⁺ cells
D
P = 0.014
ins+ cells
krt4+ traced
krt4 negative
E
P = 0.016
ins+ cells percentage
krt4+ traced
krt4 negative
F'
Number of cells
Ins+ mono
Sst1.1 mono
Ins+/Sst1.1 bi-hormonal
krt4-p2a-mNeonGreen-t2a-iCre; ubb:CSHm; Gcg, Sst1.1
30 dpf, juvenile fish
G
krt4-p2a-EGFP-t2a-CreERT2; ubb:CSHm; Gcg, Sst1.1
H
4-OHT 20 μM, 1-2 dpf

Fig. S5. Lineage-tracing experiments showing differentiation and transdifferentiation occur in parallel upon beta-cell ablation in larvae. (**A**) The workflow and time course of lineage tracing analysis of *krt4*$^{+}$ duct with MTZ treatment regimen. (**B**-**E**) Representative single-plane confocal images (**B**), quantification of Ins^{+} mono-hormonal cells, Sst1.1^{+} mono-hormonal cells and Ins^{+}&Sst1.1^{+} bi-hormonal cells in 22 principal islets in 6 dpf larvae after 2-day regeneration (**B'**), example of spatial analysis of the dorsal-ventral & anterior-posterior location (**C**), and quantification of the regenerated insulin-producing cells which can be traced back to the *krt4*$^{+}$ ductal cell origin (**D** and **E**). Scale bars = 40 µm. The white dashed lines indicate the non-Notch-responsive ductal cells. (**F**) Representative single-plane confocal image showing the long-term lineage tracing results in the principal islet with anti-Insulin and anti-Somatostatin1.1 staining in Z-projection. Quantification of Ins^{+} mono-hormonal cells, Sst1.1^{+} mono-hormonal cells and Ins^{+}&Sst1.1^{+} bi-hormonal cells in 5 principal islets in juveniles after 2-months regeneration (**F'**). Scale bars = 100 µm. (**G** and **H**) Representative confocal Z-projections with constitutive (**G**) and temporal (**H**) labeling showing the *krt4* lineage traced Gcg^{+} (yellow arrows) and Sst1.1^{+} cells (orange arrows). Scale bars = 40 µm (**G**) and 100 µm (**H**). D, dorsal; V, ventral.

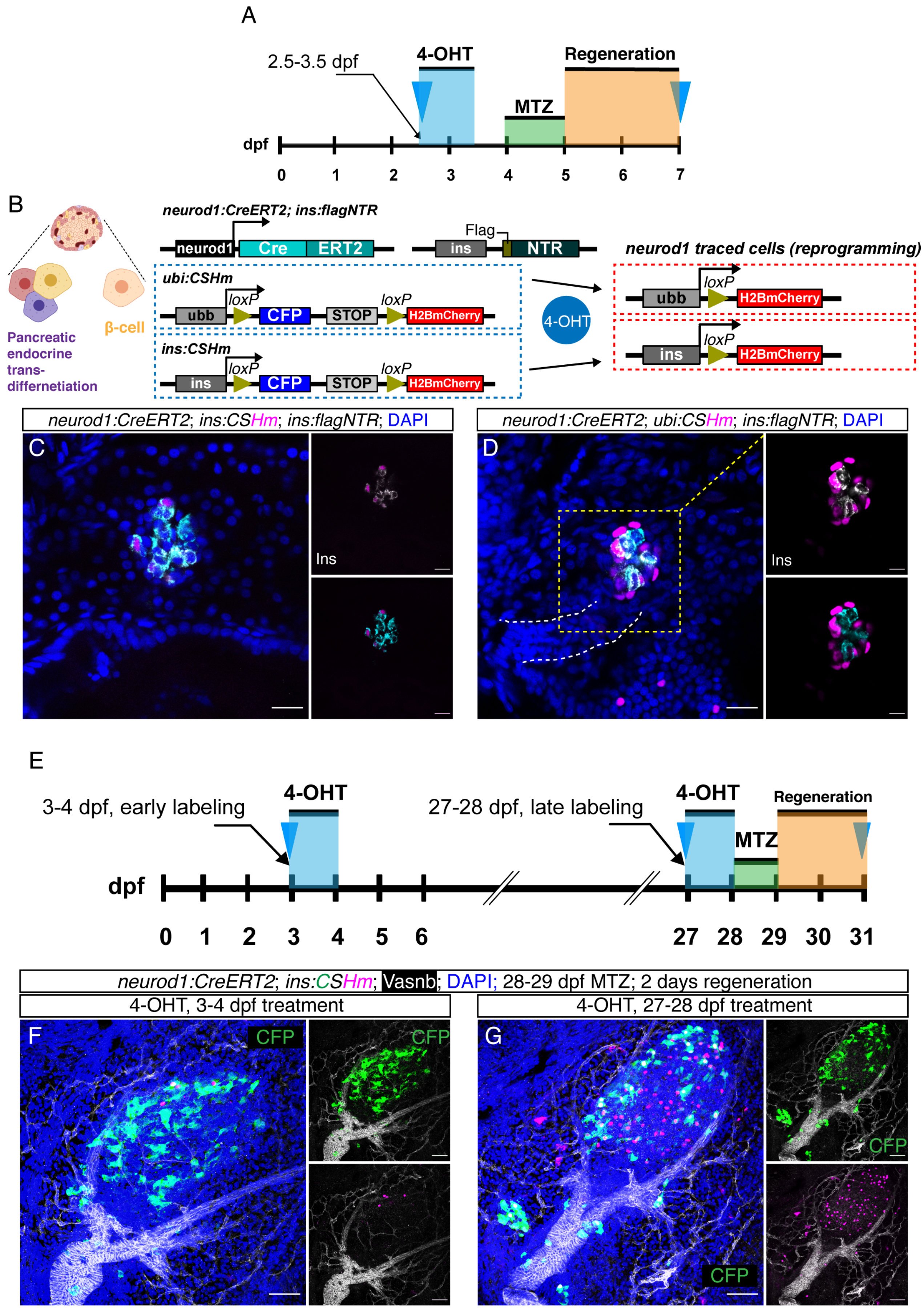
A
2.5-3.5 dpf
4-OHT
MTZ
Regeneration
dpf
0 1 2 3 4 5 6 7
B
Pancreatic endocrine trans-differentiation
β-cell
neurod1:CreERT2; ins:flagNTR
neurod1 Cre ERT2
Flag
ins NTR
ubi:CSHm
ubb loxP CFP STOP loxP H2BmCherry
ins:CSHm
ins loxP CFP STOP loxP H2BmCherry
4-OHT
neurod1 traced cells (reprogramming)
ubb loxP H2BmCherry
ins loxP H2BmCherry
neurod1:CreERT2; ins:CSHm; ins:flagNTR; DAPI
C
Ins
neurod1:CreERT2; ubi:CSHm; ins:flagNTR; DAPI
D
Ins
E
3-4 dpf, early labeling
4-OHT
27-28 dpf, late labeling
4-OHT
MTZ
Regeneration
dpf
0 1 2 3 4 5 6 27 28 29 30 31
neurod1:CreERT2; ins:CSHm; Vasnb; DAPI; 28-29 dpf MTZ; 2 days regeneration
4-OHT, 3-4 dpf treatment
4-OHT, 27-28 dpf treatment
F
CFP
CFP
G
CFP
CFP

Fig. S6. Spatiotemporal-controlled lineage tracing of *neurod1*$^+$ cells upon beta-cell ablation. (**A** and **B**) The design of the *Cre/loxP* based strategy and time course to examine whether *neurod1*$^+$ endocrine cells transdifferentiate to insulin-producing cells. (**C** and **D**) Representative single-plane confocal image showing *neurod1*$^+$ endocrine-to-insulin producing cell transdifferentiation. Fish expressing *neurod1:CreERT2* were crossed to *ins:CSHm* (**C**) or *ubi:CSHm* (**D**). The H2BmCherry expression is specifically induced in *neurod1*-derived beta-cells (**C**) or *neurod1*$^+$ cells (**D**) upon treatment of 4-OHT 2.5 μM from 2.5 to 3.5 dpf. The analyses were undertaken in 6 dpf larvae. The region outlined by the yellow square was highlighted on the right. The white dashed lines indicate the non-Notch-responsive ducts. (**E**) Experimental time course of the *Cre/loxP*-based lineage tracing experiments. The triple transgenic fish *Tg(neurod1:CreERT2);Tg(ubi:CSHm);Tg(ins:flagNTR)* are administrated 4-OHT either from 3 to 4 dpf (condition 1) or from 27 to 28 dpf, followed by 1 day MTZ treatment at day 29. Then, the juveniles were allowed to recover for 2 consecutive days and the samples were analyzed at 31 dpf. (**F** and **G**) Representative confocal Z-projections showing the contribution of regenerative *ins*$^+$ cells derived from *neurod1*$^+$ endocrine cells at 6 dpf larvae (**F**) and 31 dpf juveniles (**G**). The CFP signal was amplified by anti-GFP staining and shown in green. Scale bars = 100 μm.

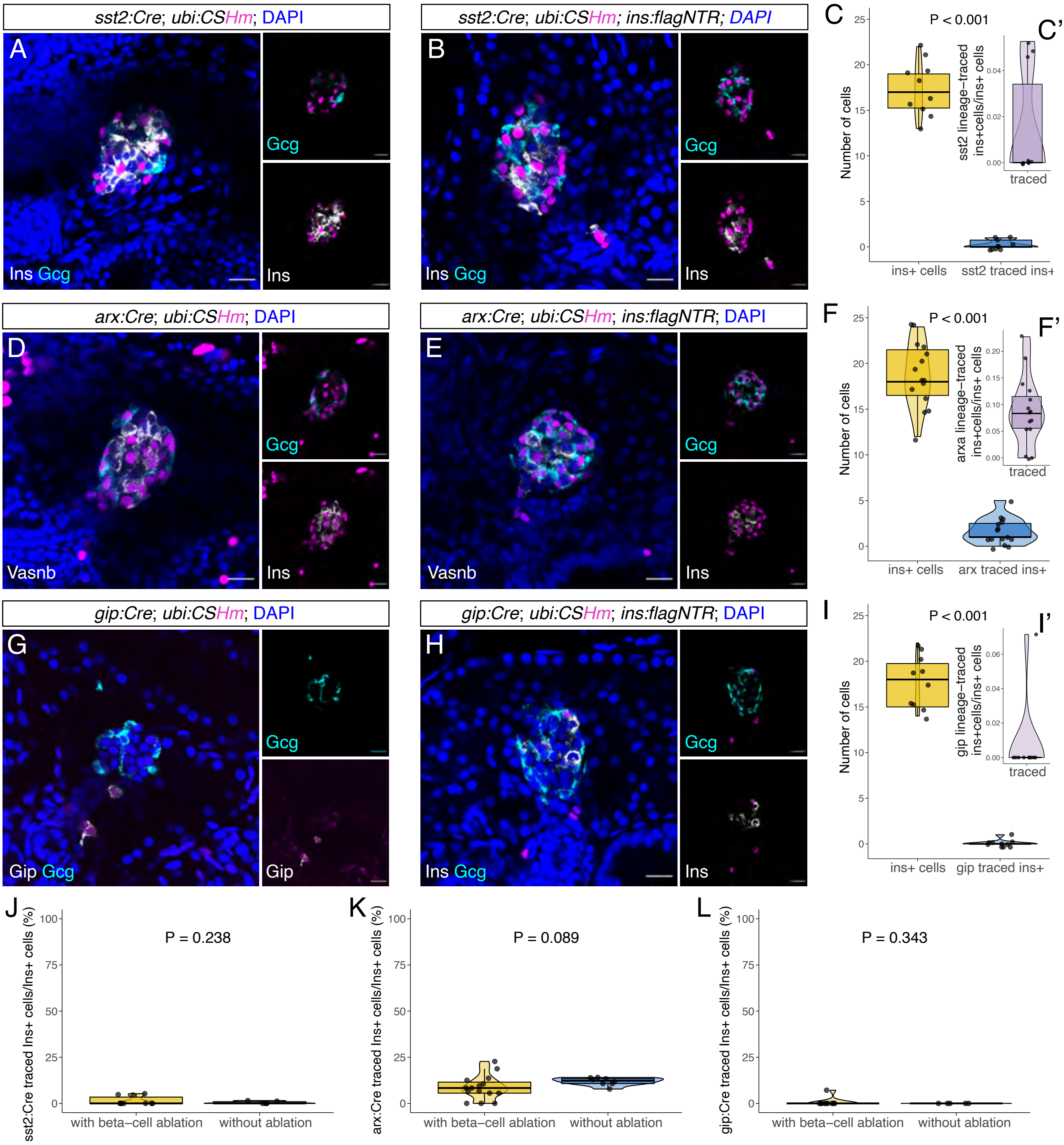
sst2:Cre; ubi:CSHm; DAPI
A
Ins Gcg
Gcg
Ins
sst2:Cre; ubi:CSHm; ins:flagNTR; DAPI
B
Ins Gcg
Gcg
Ins
C
P < 0.001
C'
Number of cells
ins+ cells
sst2 traced ins+
sst2 lineage−traced ins+cells/ins+ cells
traced
arx:Cre; ubi:CSHm; DAPI
D
Vasnb
Gcg
Ins
arx:Cre; ubi:CSHm; ins:flagNTR; DAPI
E
Vasnb
Gcg
Ins
F
P < 0.001
F'
Number of cells
ins+ cells
arx traced ins+
arxa lineage−traced ins+cells/ins+ cells
traced
gip:Cre; ubi:CSHm; DAPI
G
Gip Gcg
Gcg
Gip
gip:Cre; ubi:CSHm; ins:flagNTR; DAPI
H
Ins Gcg
Gcg
Ins
I
P < 0.001
I'
Number of cells
ins+ cells
gip traced ins+
gip lineage−traced ins+cells/ins+ cells
traced
J
sst2:Cre traced Ins+ cells/Ins+ cells (%)
P = 0.238
with beta−cell ablation
without ablation
K
arx:Cre traced Ins+ cells/Ins+ cells (%)
P = 0.089
with beta−cell ablation
without ablation
L
gip:Cre traced Ins+ cells/Ins+ cells (%)
P = 0.343
with beta−cell ablation
without ablation

Fig. S7. Lineage-tracing experiments indicate minimal contribution of insulin-producing cells originating from alpha-, delta- (*sst2*$^+$) and gip-cells. (**A**-**C**) Representative single-plane confocal images of lineage tracing of delta-cells (*sst2*$^+$) in normal (**A**) and beta-cell ablation (**B**) conditions and the quantification result depicted in the boxplots (**C** and **C'**). (**D**-**F**) Representative single-plane confocal images of lineage tracing of alpha-cells in basal state (**D**) and beta-cell ablation (**E**) conditions and the quantification result depicted in the boxplots (**F** and **F'**). (**G**-**I**) Representative single-plane confocal images of lineage tracing of gip-cells in normal (**G**) and beta-cell ablation (**H**) conditions and the quantification result depicted in the boxplots (**I** and **I'**). The quantification and comparisons of lineage traced cells using *sst2:Cre* (**J**), *arxa:Cre* (**K**), and *gip:Cre* (**L**) with or without beta-cell ablation. Scale bars = 40 μm.

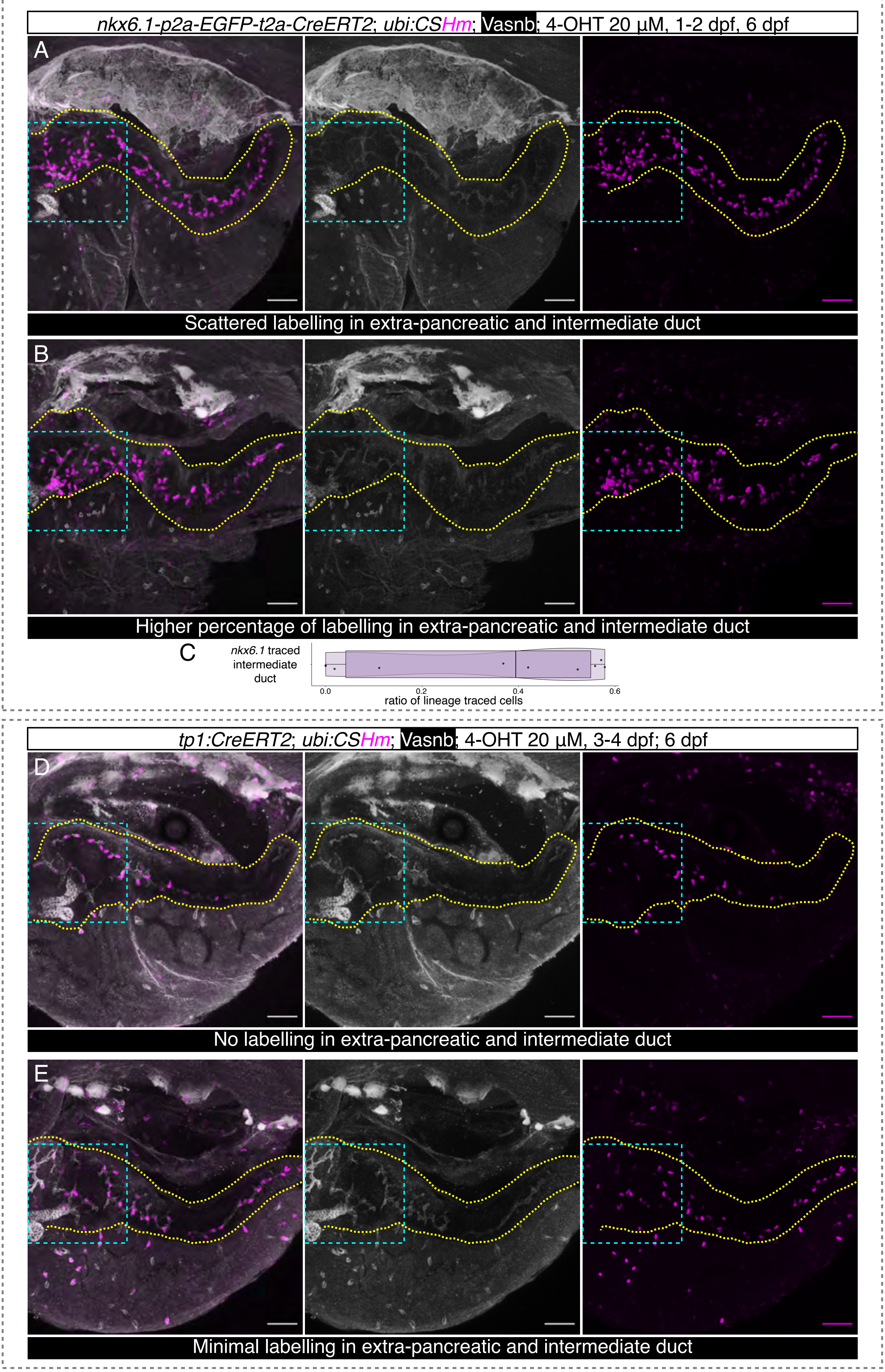
nkx6.1-p2a-EGFP-t2a-CreERT2; ubi:CSHm; Vasnb; 4-OHT 20 µM, 1-2 dpf, 6 dpf
A
Scattered labelling in extra-pancreatic and intermediate duct
B
Higher percentage of labelling in extra-pancreatic and intermediate duct
C
nkx6.1 traced intermediate duct
0.0
0.2
0.4
0.6
ratio of lineage traced cells
tp1:CreERT2; ubi:CSHm; Vasnb; 4-OHT 20 µM, 3-4 dpf; 6 dpf
D
No labelling in extra-pancreatic and intermediate duct
E
Minimal labelling in extra-pancreatic and intermediate duct

Fig. S8. Comparisons of spatiotemporal labelling of *nkx6.1*$^+$ cells and *tp1*$^+$ cells in 6 dpf larvae. (**A-C**) Confocal images displaying the variable labelling efficiency using the *nkx6.1:CreERT2* tracer in the extra-pancreatic and intermediate ductal system in Z-projection, with quantification of the proportion of labeling efficiency (**C**). (**D** and **E**) Confocal images displaying the variable labelling efficiency using *tp1:CreERT2* tracers in the extra-pancreatic and intermediate ductal system. The yellow and cyan dashed lines indicate the pancreata and the pancreatic head regions, respectively. The ductal trees were displayed by anti-Vasnb staining. Scale bars = 100 μm.

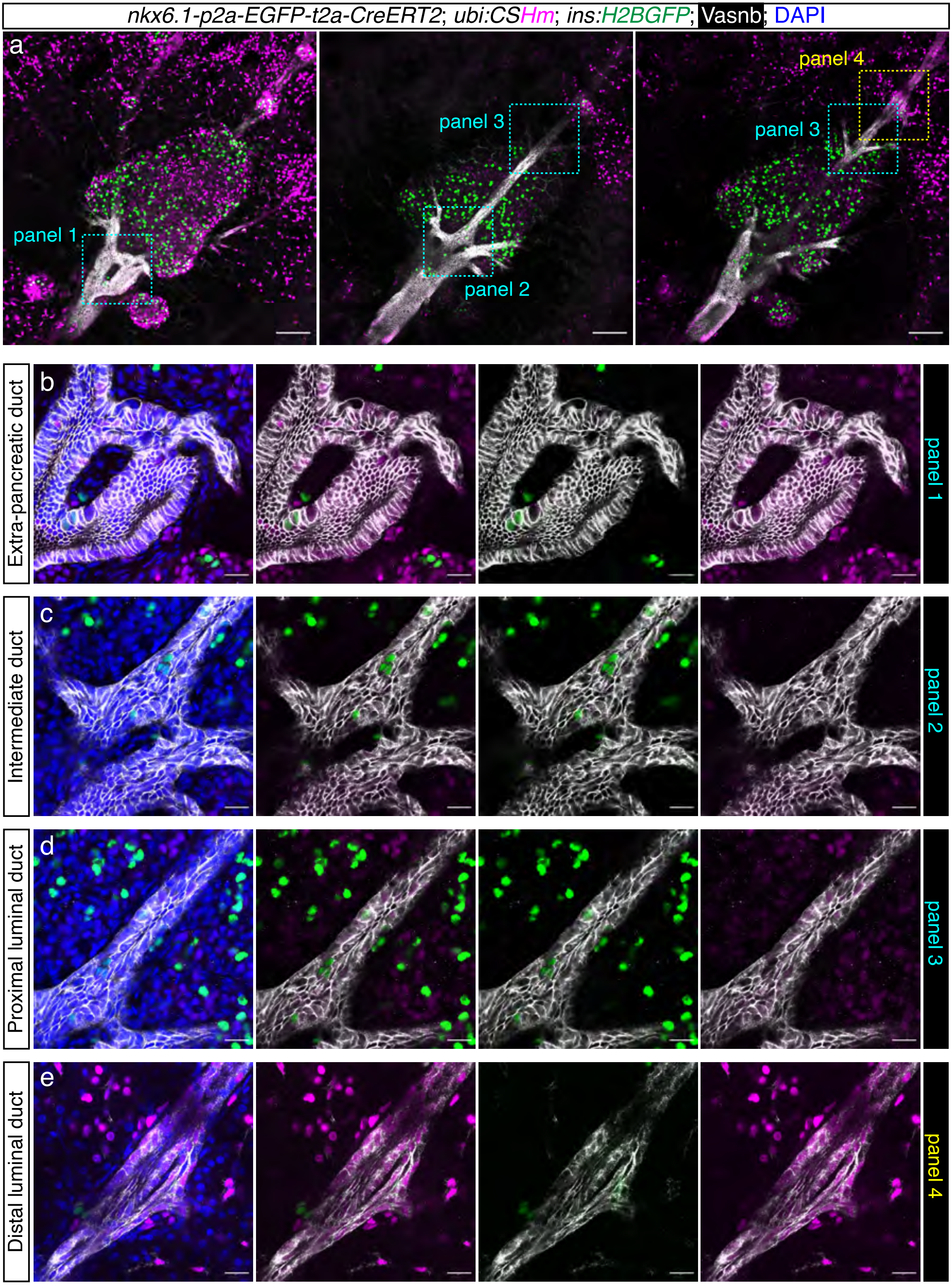

nkx6.1-p2a-EGFP-t2a-CreERT2; ubi:CSHm; ins:H2BGFP; Vasnb; DAPI
a
panel 1
panel 2
panel 3
panel 3
panel 4
b
c
d
e
Extra-pancreatic duct
Intermediate duct
Proximal luminal duct
Distal luminal duct
panel 1
panel 2
panel 3
panel 4

Fig. S9. Spatiotemporal-controlled lineage tracing of *nkx6.1*$^{+}$ ducts with a subset of extra-pancreatic duct labelled. (**A**) Single-plane confocal images showing the labelling pattern of three distinct regions with high-magnifications shown respectively in (**B**-**E**). A subset of extra-pancreatic duct and intermediate ducts can be traced back to *nkx6.1*$^{+}$ cell origin, while the ductal cells residing in between the intermediate duct and the distal luminal duct in the tail regions are devoid of labelling. The distal luminal ducts in the pancreatic tail are H2BmCherry positive. Anti-Vasnb staining (white) and the *ins:H2BGFP* transgene (green) were utilized to locate ductal trees and islets. Scale bars = 100 μm (**A**) and 40 μm (**B**-**E**).

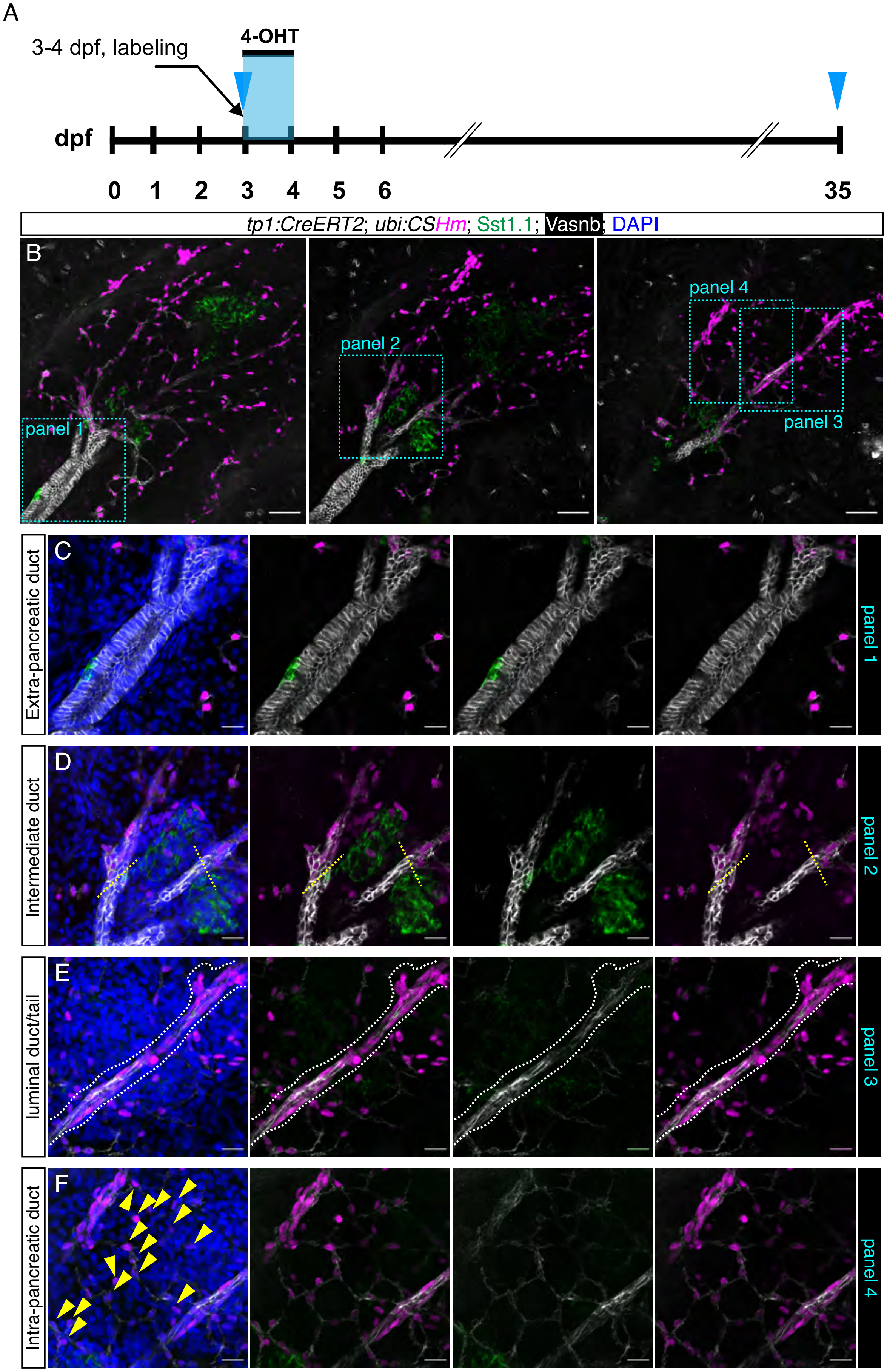

A
3-4 dpf, labeling
4-OHT
dpf
0
1
2
3
4
5
6
35
tp1:CreERT2; ubi:CSHm; Sst1.1; Vasnb; DAPI
B
panel 1
panel 2
panel 4
panel 3
C
Extra-pancreatic duct
panel 1
D
Intermediate duct
panel 2
E
luminal duct/tail
panel 3
F
Intra-pancreatic duct
panel 4

Fig. S10. Spatiotemporal-controlled lineage tracing showing the progenies of *tp1* driven Notch-responsive duct. (**A**) Experimental time course of the *Cre/loxP* system used for spatiotemporal lineage tracing of $tp1^+$ Notch-responsive ductal cells. The H2BmCherry expression is specifically induced in in $tp1^+$ ducts and their progenies by 20 μM 4-OHT application from 3 to 4 dpf. The analysis was made at 35 dpf. (**B**-**F**) Single-plane confocal images showing the labelling pattern of four distinct regions with high-magnifications shown respectively in (**C**-**F**). The ducts below the yellow dashed lines are intermediate duct (**D**). Extra-pancreatic ducts and intermediate ducts are devoid of labelling while the luminal ducts that appear in the pancreatic tail regions are H2BmCherry positive, indicating a Notch-responsive duct origin. The dashed line highlights the luminal duct (**E**) and the arrows point to the intra-pancreatic duct (**F**). Anti-Vasnb (white) and anti-Somatostatin1.1 staining (green) were utilized to locate ductal tree and islets. Scale bars = 100 μm (**B**) and 40 μm (**C**-**F**).

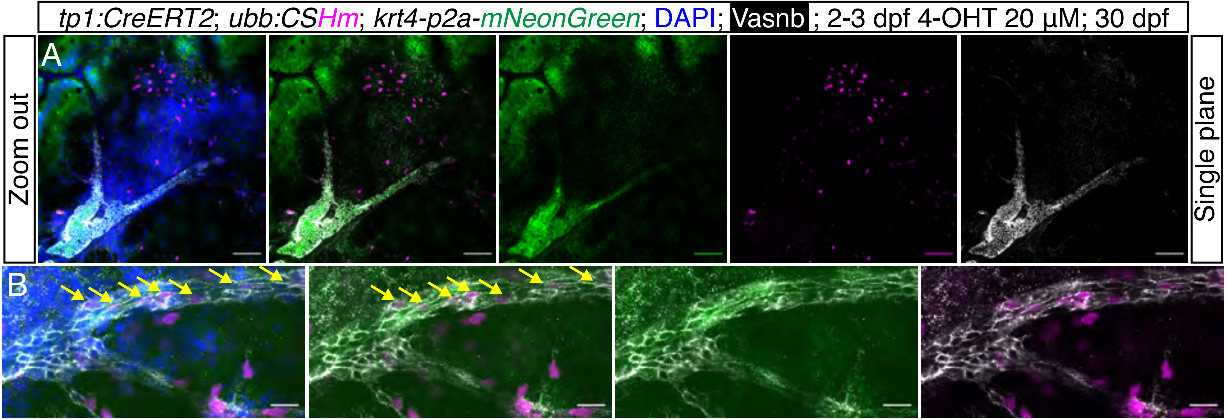
tp1:CreERT2; ubb:CSHm; krt4-p2a-mNeonGreen; DAPI; Vasnb ; 2-3 dpf 4-OHT 20 μM; 30 dpf
Zoom out
A
Single plane
B

Fig. S11. Spatiotemporal-controlled lineage tracing showing the progenies of *tp1* derived intra-pancreatic duct remodeling into *krt4*+ luminal duct. (**A and B**) Single-plane confocal images showing the labelling pattern in both pancreatic head (A) and magnification in the tail region (B). The yellow arrows pointed to *tp1* lineage traced cells that reside within *krt4*+ luminal duct indicated by mNeonGreen and Vasnb staining. Scale bars = 100 μm (**A**) and 40 μm (**B**).

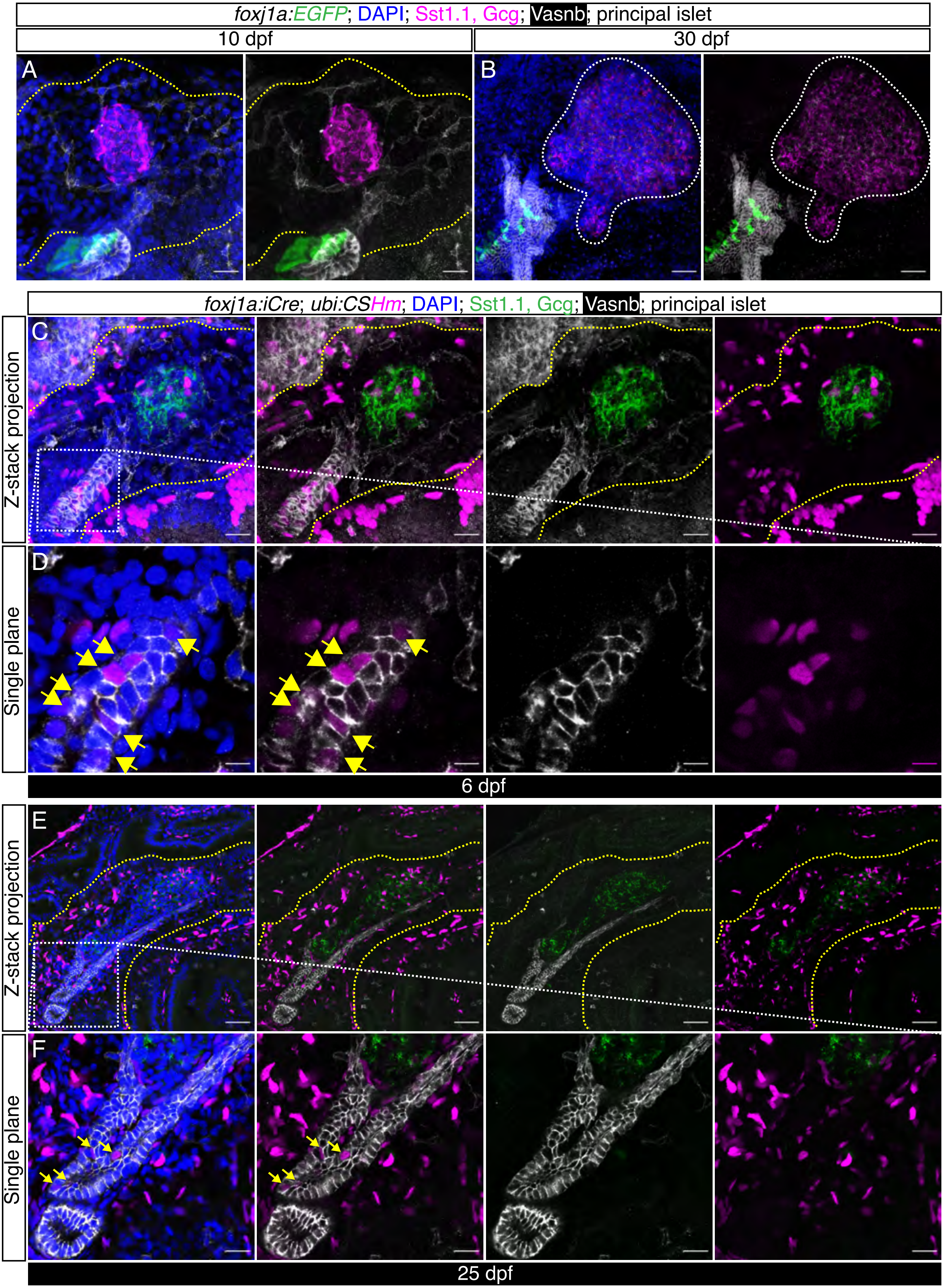

foxj1a:EGFP; DAPI; Sst1.1, Gcg; Vasnb; principal islet
10 dpf
30 dpf
A
B
foxj1a:iCre; ubi:CSHm; DAPI; Sst1.1, Gcg; Vasnb; principal islet
Z-stack projection
C
Single plane
D
6 dpf
Z-stack projection
E
Single plane
F
25 dpf

Fig. S12. Long-term lineage tracing of *foxj1a*⁺ cells. (**A** and **B**) Representative confocal Z-projections of *foxj1a*$^+$ cells in 10 and 30 dpf juveniles depicted in *foxj1a:EGFP* transgenics. The white and yellow dashed lines indicate the pancreata and the principal islet, respectively. (**C**-**F**) Lineage-tracing experiments showing the *foxj1a*$^+$ lineage in a 6 dpf larva (**C** and **D**) and 25 dpf juvenile fish (**E** and **F**). The high magnification of the region showing mosaic labeling of the extra-pancreatic duct and the absence of labelling of intermediate duct (**C** and **E**). The yellow dashed lines indicate the pancreata. The yellow arrows point to *foxj1a*-lineage traced cells residing in the extra-pancreatic duct.

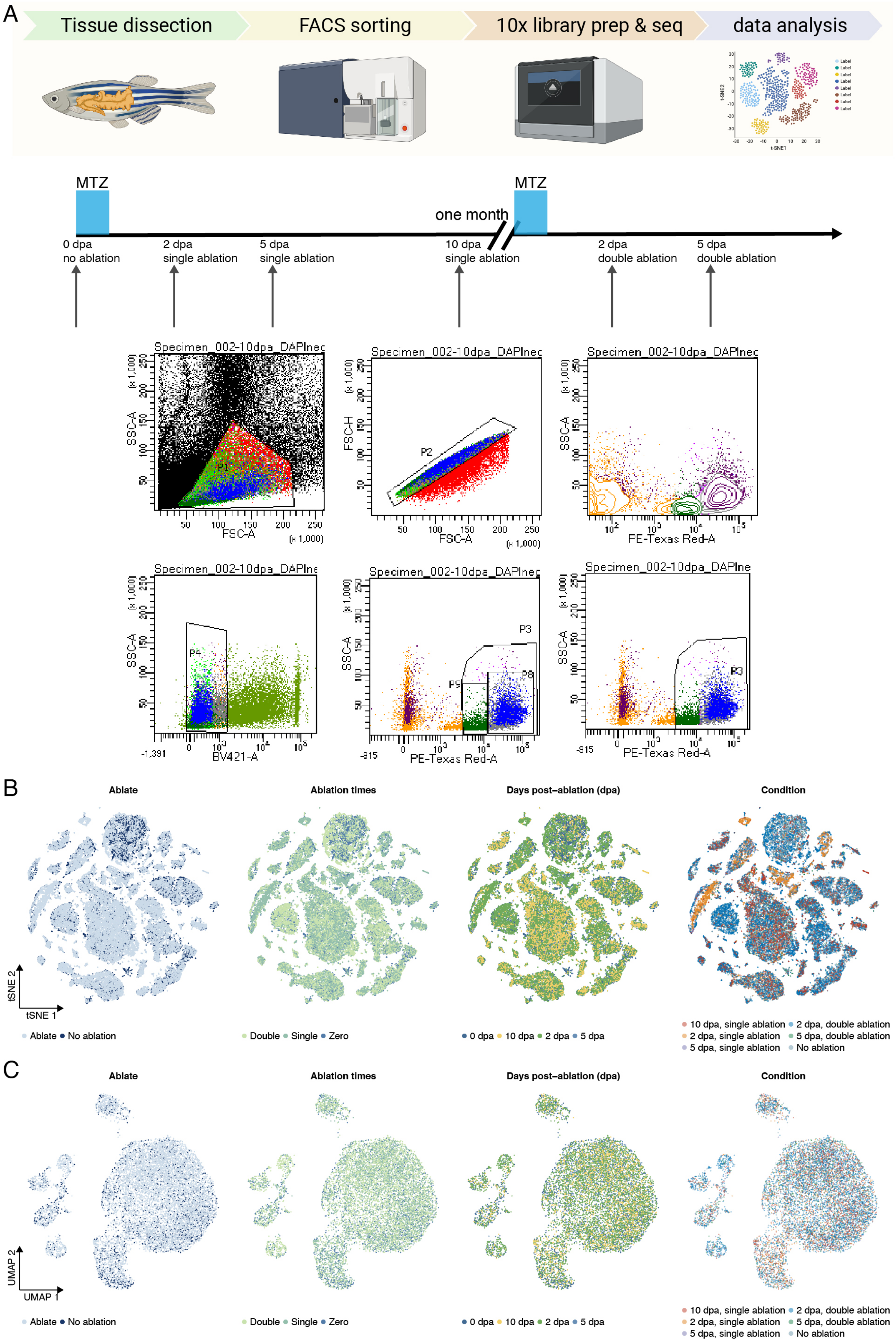

A
Tissue dissection
FACS sorting
10x library prep & seq
data analysis
MTZ
MTZ
one month
0 dpa
no ablation
2 dpa
single ablation
5 dpa
single ablation
10 dpa
single ablation
2 dpa
double ablation
5 dpa
double ablation
Specimen_002-10dpa_DAPIneg
SSC-A
FSC-A
FSC-H
P1
P2
P3
P4
P8
P9
PE-Texas Red-A
BV421-A
B
Ablate
Ablation times
Days post–ablation (dpa)
Condition
tSNE 2
tSNE 1
Ablate
No ablation
Double
Single
Zero
0 dpa
10 dpa
2 dpa
5 dpa
10 dpa, single ablation
2 dpa, double ablation
2 dpa, single ablation
5 dpa, double ablation
5 dpa, single ablation
No ablation
C
UMAP 2
UMAP 1

Fig. S13. The pipeline of single-cell RNA-seq. (**A**) Schematic diagram of the single-cell RNA-seq pipeline of *krt4*$^{+}$ cells in the zebrafish pancreas, including tissue processing, fluorescence-activated cell sorting (FACS) and scRNA-seq profiling. The pancreata were dissected out carefully from *TgKI(krt4:iCre);Tg(ubi:CSHm);Tg(ins:flagNTR)* adult (6-9 months old) with minimum contamination by carefully removing skin, intestine, liver and gallbladder. Up to eight samples from each condition were pooled together and enzymatic dissociated into single-cell suspension. Single mCherry^{+}/DAPI^{-} cells were sorted (shown in representative FACS plot) and followed by 10x Genomics library preparation and Next-generation sequencing. (**B** and **C**) UMAP visualization of 10 single-cell datasets colored by batch conditions, with or without ablation, and ablation times.

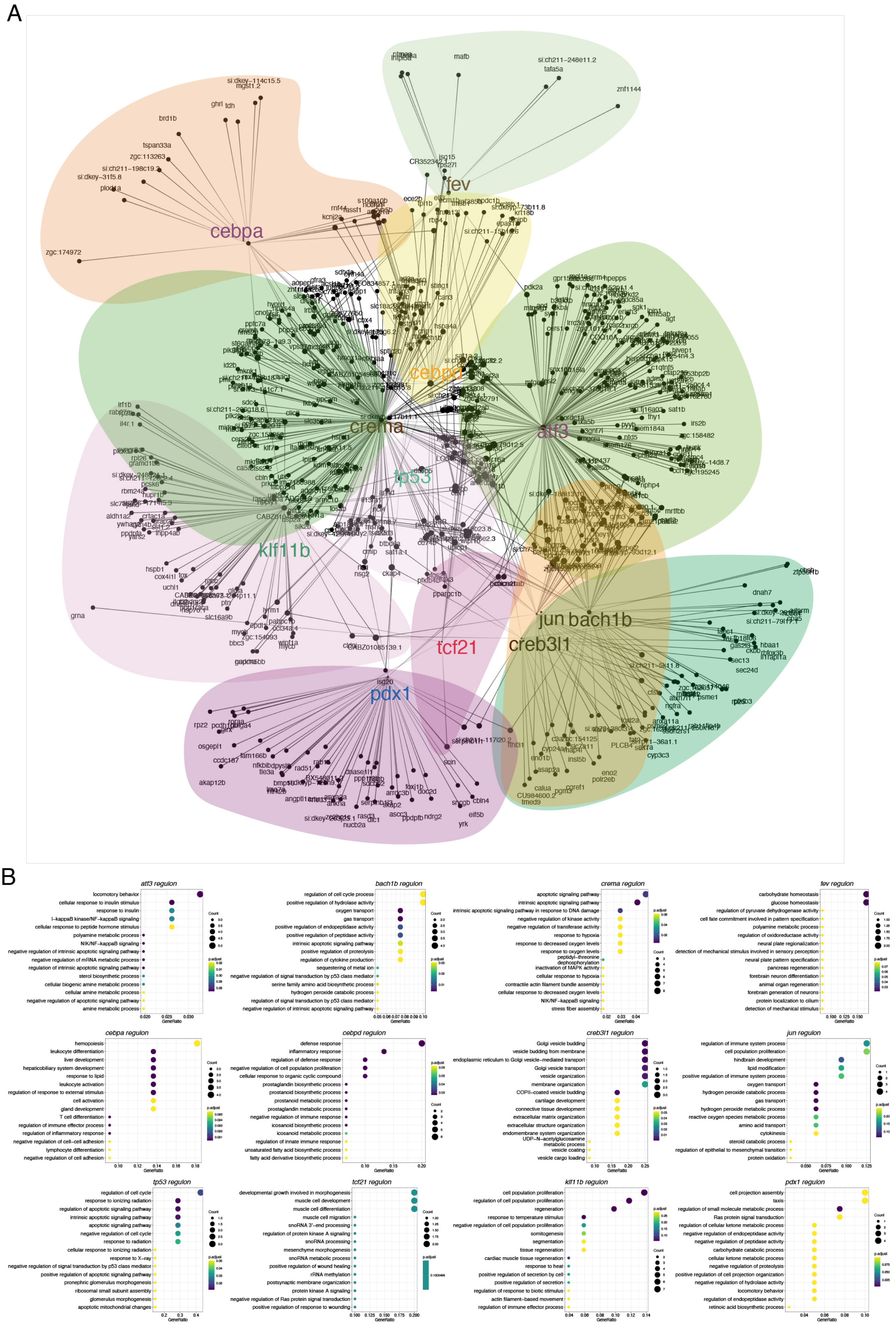

A
cebpa
fev
cebpd
crema
atf3
tp53
klf11b
jun
bach1b
creb3l1
tcf21
pdx1
B
atf3 regulon
bach1b regulon
crema regulon
fev regulon
cebpa regulon
cebpd regulon
creb3l1 regulon
jun regulon
tp53 regulon
tcf21 regulon
klf11b regulon
pdx1 regulon
GeneRatio
Count
p.adjust

Fig. S14. The connectivity and GO enrichment of regulons. (**A**) The intramodular regulatory networks were predicted using SCENIC pipeline and viewed by graph. The connections indicate statistically significant regulations with colors representing each regulon. The outlines were colored by the regulons. (**B**) Dot plots showing GO results of target gene sets for each regulon, colored by adjusted p-value and sized by the number of genes.

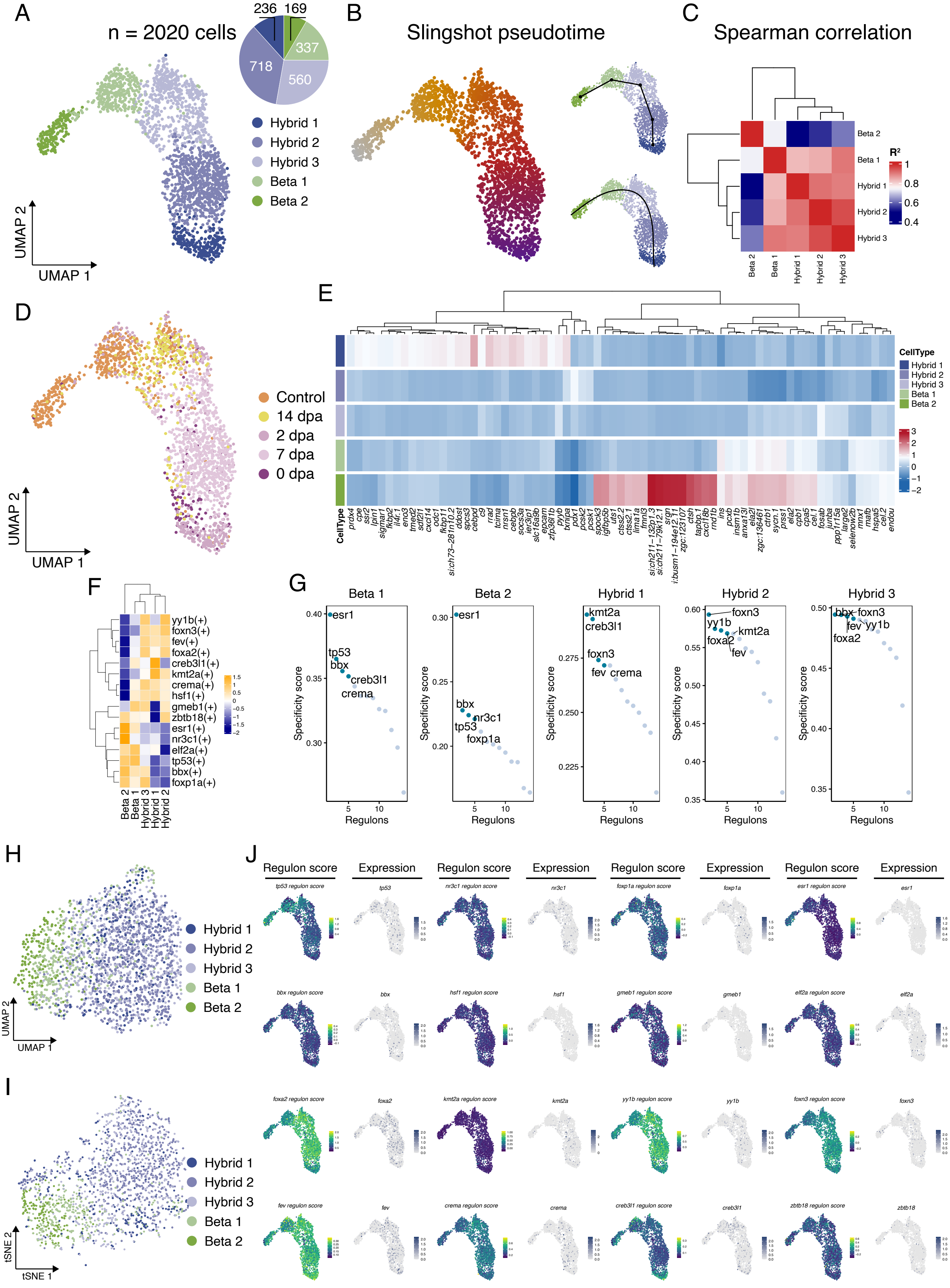

A
n = 2020 cells
236
169
337
718
560
Hybrid 1
Hybrid 2
Hybrid 3
Beta 1
Beta 2
UMAP 2
UMAP 1
B
Slingshot pseudotime
C
Spearman correlation
Beta 2
Beta 1
Hybrid 1
Hybrid 2
Hybrid 3
R²
D
Control
14 dpa
2 dpa
7 dpa
0 dpa
E
CellType
F
G
Beta 1
Beta 2
Hybrid 1
Hybrid 2
Hybrid 3
Specificity score
Regulons
H
I
tSNE 2
tSNE 1
J
Regulon score
Expression

Fig. S15. *in silico* analyses of *ins*$^{+}$/*sst1.1*$^{+}$ hybrid cell-to-beta cell trajectory. (**A**) UMAP plot displaying 5 cell clusters of *ins*$^{+}$/*sst1.1*$^{+}$ hybrid cell-to-beta cells, color-coded by subpopulation. The numbers and proportions of each cell type were displayed in a piechart. (**B**) Slingshot analysis reveals a linear trajectory. (**C**) The heatmap showing the Spearman's correlation based on the highly variable genes. (**D**). The same UMAP overlayed with sample batch information. (**E**) Heatmap of the top differentially expressed genes per cluster, with selected markers gene. (**F**) Heatmaps showing the enriched regulons among the 5 cell states. The regulons were ordered by hierarchical clustering. (**G**) The dot plot visualizes the highly enriched regulon for each cell state. (**H**) The UMAP plot based on the regulon activity scores (RAS), each cell is color-coded based on the cell states assignment. (**I**) The t-SNE plot based on the regulon activity scores (RAS), each cell is color-coded based on the cell states assignment. (**J**) UMAP projection overlayed by the expression level of transcriptional factors (right) and RAS (left).
